# Spatio-temporal Organization of Network Activity Patterns in the Hippocampus

**DOI:** 10.1016/j.celrep.2025.115808

**Published:** 2025-06-04

**Authors:** Vítor Lopes-dos-Santos, Demi Brizee, David Dupret

**Affiliations:** 1 Medical Research Council Brain Network Dynamics Unit, Nuffield Department of Clinical Neurosciences, University of Oxford, Oxford OX1 3TH, UK

## Abstract

Understanding how coordinated neural networks support brain functions remains a central goal in neuroscience. The hippocampus, with its layered architecture and structured inputs to diverse cell populations, is a tractable model for dissecting operating microcircuits through the analysis of electrophysiological signatures. We investigated hippocampal network patterns in behaving mice by developing a low-dimensional embedding of local field potentials recorded along the CA1-to-dentate gyrus axis. This embedding revealed layer-specific gamma profiles reflecting spatially organized rhythms and their associated principal cell-interneuron firing motifs. Moreover, firing behaviors along the CA1 radial axis distinguished between deep and superficial principal cells, as well as between interneurons from the pyramidal, radiatum, and lacunosum-moleculare layers. These findings provide a comprehensive map of spatio-temporal activity patterns underlying hippocampal network functions.

## Introduction

Brain networks consist of diverse neuronal populations organized into intricate microcircuits. Understanding the mechanisms that enable their coordinated activity to support network functions remains a central goal in neuroscience. Electrophysiology, combined with anatomical insights, is a powerful approach to achieve this objective. However, fully characterizing and interpreting the electrical signatures emanating from these networks remains a key challenge.

The hippocampus is a network at the nexus of the brain’s circuitry for internal information processing^1–4^. Its primary output – the CA1 pyramidal cells – express diverse firing patterns shaped by inputs from within and outside the hippocampus and refined by local interneurons^5–8^. These inputs and interneurons are organized in layers along the pyramidal cells’ somato-dendritic axis^6,9–11^. This radial arrangement produces a rich repertoire of electrophysiological patterns in the local field potentials (LFPs). Deepening our understanding of these signatures could clarify the processes underlying hippocampal functions.

Hippocampal network patterns are behavioral state dependent. Sharp-wave ripples (SWRs) in the *Cornu Ammonis* (CA) and dentate spikes in the dentate gyrus (DG) occur during sleep and immobility. Dentate spikes are large-amplitude events observed in DG LFPs, linked to entorhinal cortex inputs to DG moleculare layers^12–17^. The SWR complex originates from a current sink in the CA1 radiatum (the sharp-wave), driven by CA3 inputs, and culminates in brief high-frequency (100 – 250 Hz) oscillations (the ripples) in the CA1 pyramidal layer^18–20^. Deciphering the mechanisms underlying these phenomena has led to significant insights into the role of SWRs in memory stabilization and learning^21–30^.

During active exploration, the rodent hippocampus exhibits theta (5 – 12 Hz) oscillations^31,32^, which coordinate hippocampal circuits and orchestrates interactions with extrahippocampal regions^33–37^. Theta oscillations nest gamma rhythms (25 – 150 Hz), bursts of rhythmic activity reflecting circuits and pathways across the hippocampal formation^38–43^. Gamma oscillations serve as proxies for the activity of known pathways and microcircuits, enabling the study of their functions^44–49^. Identifying novel gamma oscillations may reveal neuronal motifs not evident from anatomy alone.

The radial organization of hippocampal microcircuits encompasses substantial neuronal diversity. Until recently, CA1 principal cells were assumed to form a homogeneous population. However, recent research has identified two parallel microcircuits among CA1 principal cells, with distinct properties and connectivity, supporting complementary processing channels^50–54^. While CA1 principal cells are densely packed within the pyramidal layer, interneurons are dispersed across layers^55^. These include multiple cell types that target different pyramidal cell subdomains, display distinct firing patterns, and likely serve specialized roles in the local CA1 network^10,56,57^. Whether interneuron soma location relates to their electrophysiological heterogeneity remains unclear.

We explored the temporal architecture of hippocampal activity across layers by developing a low-dimensional embedding representing electrophysiological patterns from behaving mice implanted with silicon probes. Individual LFPs along the CA1-to-DG axis carried sufficient information within theta and sharp-wave waveforms to distinguish hippocampal layers. Each discerned layer displayed a distinct theta-nested gamma profile, reflecting the coexistence of multiple hippocampal gamma oscillations. In subsequent experiments, the embedding guided the placement of independently movable tetrodes in specific CA1 layers. Individual adjustment of tetrodes allowed maximizing single unit yield and target interneurons in the sparse stratum radiatum and lacunosum-moleculare layers. Analyses of gamma-related firing revealed rhythm-specific principal cell-interneuron temporal motifs. Further analysis of spiking across layers uncovered layer-specific dynamics, differentiating deep and superficial pyramidal cells, as well as neurons in the radiatum versus lacunosum-moleculare layers. Together, these findings highlight the fine-grained spatio-temporal organization of hippocampal network activity.

## Results

### Identifying individual hippocampal layers along the CA1-to-DG axis

**Figure 1:**
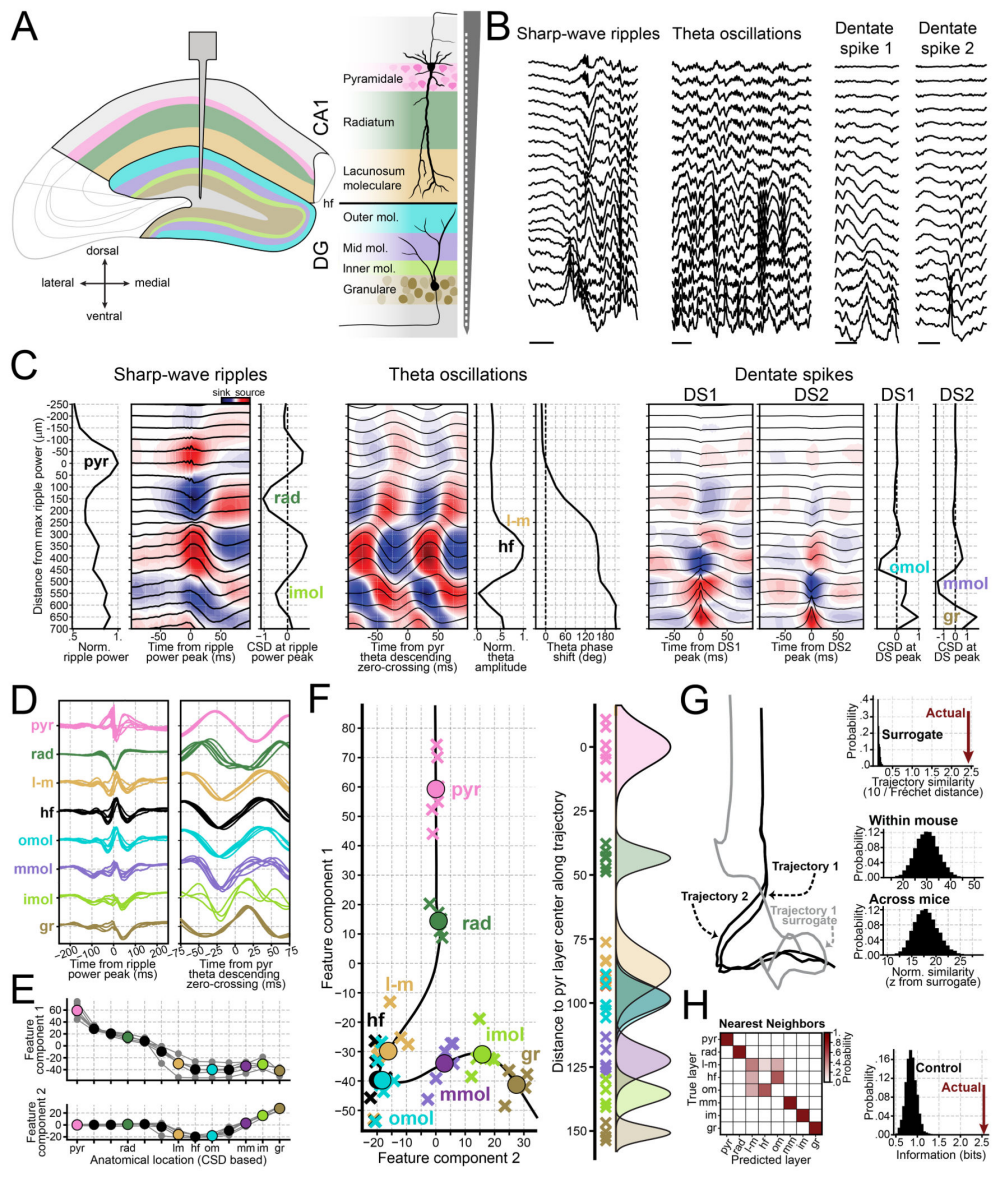
Identification of hippocampal layers using electrophysiological patterns. (A) Schematic of the hippocampal layers recorded using a silicon probe implanted along the CA1-DG axis. (B) Sample data showing the three network events used for layer identification. Scale bars: 100 ms. (C) Example activity profiles for layer determination (Mouse 1; see Figure S1 for other mice). The sharp-wave ripple panels display ripple power across layers, CSD analysis of LFPs aligned to ripple power peaks, and CSD values at the ripple power peak. The theta oscillation panels display the CSD aligned to the pyramidal theta descending zero-crossing, alongside normalized theta amplitude and phase shift across layers. The dentate spike panels depict CSD analysis for DS1 and DS2, with their respective CSD values at their peaks. (D) Sharp-wave and theta waveforms for CSD-defined layers (one waveform per mouse). (E) Construction of the embedding trajectory. The sharp-wave and theta waveforms recorded across layers were used to generate the embedding. Shown are the two ISOMAP components. To compute a trajectory, embedding coordinates from each layer and additional intermediate points were averaged across mice. Gray lines represent individual mice; color-coded circles show the across-mice average per layer; black circles denote intermediate points. The trajectory was defined by interpolating between the average coordinates. (F) Left: LFP-based feature embedding. Each cross represents one mouse and is color-coded as in (D); circles indicate across-mice layer averages. The trajectory is shown as a black trace. Right: Linearized representation of the trajectory. (G) Trajectory consistency analysis. Trajectory similarity was quantified via Fréchet distances. Distances were computed across sessions within a mouse or across different mice. Left: Example of two actual trajectories from different mice and a corresponding surrogate. Top right: Trajectory similarity for a representative pair along with its surrogate distribution. Bottom right: Bootstrap distributions for normalized similarity (z-scored using the surrogate distribution’s mean and standard deviation) for within-mouse and across-mice pairs. (H) Left: Confusion matrices for a classifier predicting the layer from feature space coordinates. Matrix entries represent the likelihood of predicting a specific layer given the true layer. Right: Mutual information between the actual and the predicted layers, compared to a control distribution obtained by shuffling layer labels. Abbreviations: *pyr*, pyramidale; *rad*, radiatum; *l-m*, lacunosum-moleculare; *hf*, hippocampal fissure; *omol*, outer molecular; *mmol*, mid molecular; *imol*, inner molecular; *gr*, granular.

We tested whether LFP patterns could distinguish the radially organized layers from CA1 to DG in the dorsal hippocampus. We implanted linear silicon probes in six mice (Table S1) along the somato-dendritic axis of CA1 principal cells, extending up to the upper DG blade (Figure 1A). This setup allowed simultaneous LFP recordings across layers during open-field exploration and sleep/rest. We focused on three electrophysiological patterns (Figure 1B): theta oscillations (5 – 12 Hz) during exploration, as well as sharp-wave ripples (SWRs) and dentate spikes (DS1 and DS2) during sleep/rest.

Anatomical layers were identified along the probe using established electrophysiological signatures (Figure 1C). The CA1 pyramidal layer channel was identified as the one with the highest ripple power, while the radiatum was identified by the strongest current sink coinciding with the ripple amplitude peak^20,58–60^. The hippocampal fissure channel was identified as the channel with the highest theta power^61–64^, while the lacunosum-moleculare was defined as being 40–50 μm above the fissure.

In DG, the outer and mid-molecular layers corresponded to the deepest current sinks for DS1 and DS2 events, respectively^12,59^. The inner molecular layer was identified by a secondary current sink associated with the sharp-wave event^18,59^. The granular layer was identified by the strongest current source of DS beneath the molecular layers^12,16,17,59^. These spatio-temporal patterns were consistent across mice (Figure S1).

This layer assignment was facilitated by silicon probes, which enabled recordings from uniformly spaced channels along the CA1-DG axis. We next tested whether layer identity could be inferred from theta phase reversals and sharp-wave waveform features on a per-channel basis. To this end, we applied ISOMAP embedding to represent theta and sharp-wave waveforms across layers in a two-dimensional feature space (Figure S2 and Figure 1D-F). Channels from the same layer clustered together across mice (Figure 1F). The resulting feature trajectory naturally connected anatomically adjacent layers, despite no anatomical information provided to the embedding.

To assess trajectory consistency across recordings, we computed their similarity and compared it to surrogate trajectories that preserved the original spectral properties (Figure 1G). For recordings from the same mouse across days, similarity was 30.1 standard deviations above the surrogate expected values (99% CI: 18.1–42.9). Across mice, similarities remained 18.0 standard deviations above the control values (99% CI: 12.2–24.8). This demonstrates that trajectories were highly reproducible within and across mice.

To further assess the electrophysiological distinguishability of hippocampal layers we trained classifiers to recognize each layer from its embedding projections using leave-one-out cross-validation (Figure 1H). Classification was optimal for the pyramidal, radiatum, mid-molecular, and granular layers. The inner molecular layer achieved near-optimal performance. The lacunosum-moleculare layer, hippocampal fissure, and outer molecular layer were differentiated from other layers; yet, splitting these three layers remained challenging (Figure 1H; left). To quantify layer-specific information in the feature space, we calculated the Shannon information between actual and predicted layers. These values were significantly higher than those obtained from controls with shuffled labels (Figure 1H; right). These findings demonstrate that LFP-based landmarks are consistent across mice and enable reliable layers identification along the CA1-DG axis.

### Theta-nested gamma oscillations along the CA1-to-DG laminar profile

**Figure 2:**
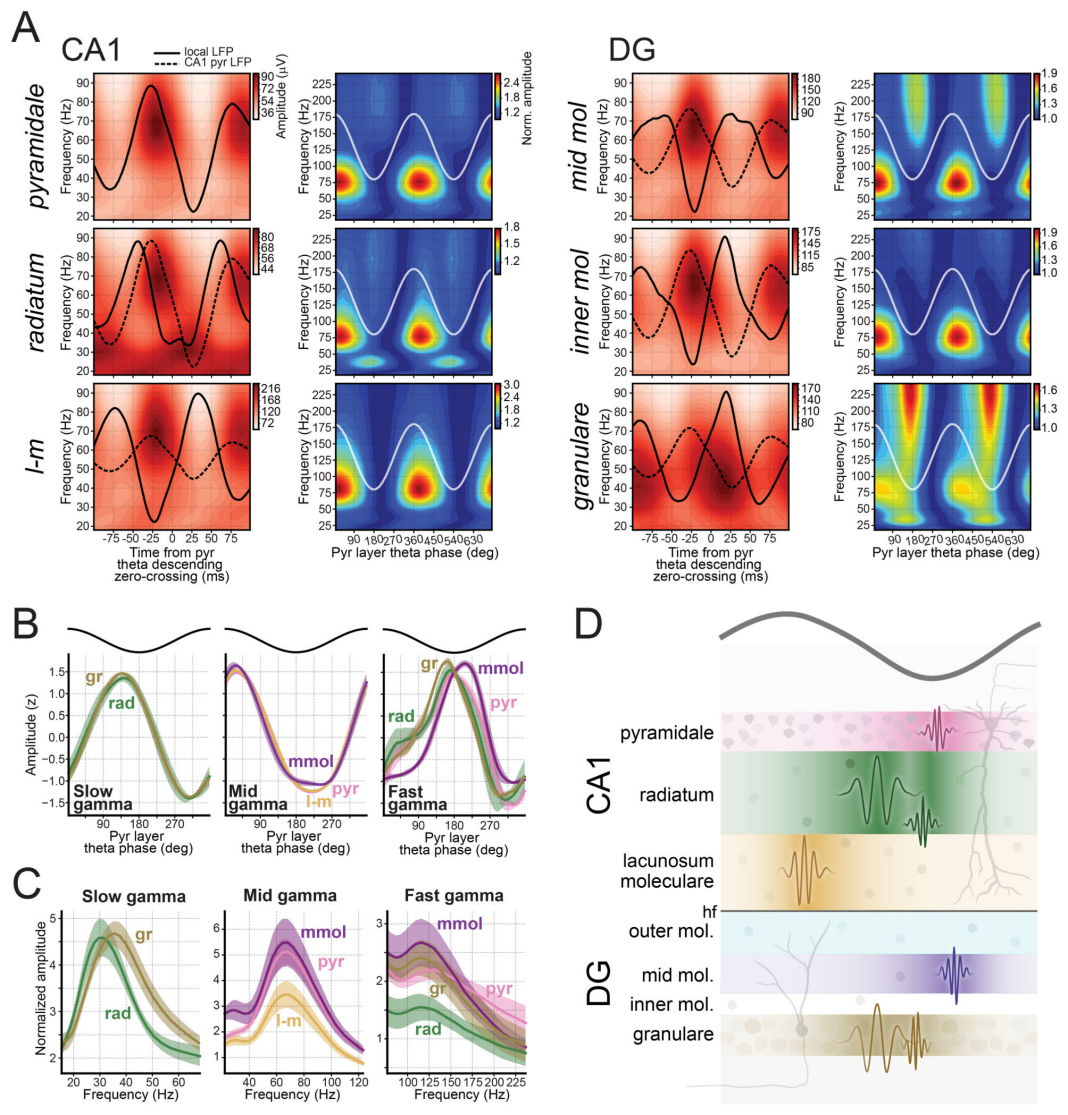
Theta-nested gamma profiles across individual CA1-to-DG layers. (A) Each layer is represented by two panels displaying theta-nested gamma amplitudes. Left panels show both local (solid lines) and CA1 pyramidal layer LFPs (dashed lines), aligned to the descending zero-crossing of the pyramidal layer theta. These are overlaid on the gamma-frequency amplitudes. Right panels show each frequency’s amplitude normalized to its minimum value. Data are from a representative mouse (see Figure S3A for other mice). (B) Z-scored amplitudes for gamma bands across layers relative to the pyramidal layer theta phase. (C) Normalized power spectra calculated for each gamma rhythm in specific layers. In both (B) and (C), solid lines denote the mean across mice, and shaded regions indicate 95% confidence intervals. (D) Schematic illustrating gamma oscillations across hippocampal layers. Each gamma oscillation is placed at the theta phase where it reaches its maximum amplitude. The mid-gamma rhythm (dark orange) appears in all layers but is shown in the lacunosum-moleculare layer, where its amplitude is strongest.

Theta oscillations influence large-scale brain networks, while hippocampal gamma oscillations reflect fine-grained pathways^38–40^. Analyzing gamma across layers could thus clarify the functional microarchitecture of hippocampal networks. We generated theta-gamma profiles for each layer distinguishable in the embedding space (Figure 1F,H) using two complementary evaluations (Figures 2A and S3A). First, we computed the amplitude of individual gamma frequencies using the CA1 pyramidal theta rhythm as a phase reference (Figure 2A; left panel for each layer). This enabled comparing amplitudes of gamma frequencies within each layer. Second, we normalized each frequency’s amplitude by its minimum value (Figure 2A; right panel for each layer), rendering a perspective on gamma amplitude modulation by theta phase.

In CA1, mid gamma (~50 – 90 Hz) oscillations were present across all layers (Figure 2A), with their maximum amplitude occurring just after the pyramidal layer theta peak (Figure 2A,B). The pyramidal layer also showed a fast gamma component (~100 – 250 Hz) increasing at the theta trough^41,42,45,46^ (Figures 2A,B and S3A), though with lower amplitude than mid gamma. The radiatum layer displayed slow gamma oscillations (~25 – 45 Hz) at the descending theta phase, alongside a fast gamma component (Figure 2A,B). While slow gamma in radiatum has been described previously^41,42,45,46,65^, this radiatum fast gamma has not been reported. The lacunosum-moleculare profiles showed mid gamma oscillations but lacked clear slow or fast gamma components (Figure 2A). These findings were reproducible across mice (Figure S3A).

In DG, two distinct fast gamma components were observed: one in the mid-molecular layer just after the pyramidal theta trough, and another in the granular layer just before the theta trough (Figures 2A,B and S3A). The granular layer also displayed a slow gamma component, aligned with that observed in radiatum (Figure 2A,B and S3A). Like CA1, all DG layers displayed mid gamma oscillations. Additionally, a beta component (~18 – 35 Hz) was observed in the mid-molecular layer, reaching its maximum amplitude just after the pyramidal theta peak (Figure S3A). Although occupying lower frequencies, this beta component emerged at a distinct theta phase from slow gamma.

Assigning precise frequency ranges to gamma oscillations is challenging–especially for fast gamma, which has significantly lower amplitudes than mid gamma oscillations (Figure 2A) and can be overshadowed in non-normalized spectrograms. Conversely, normalized spectrograms can distort the frequency range of oscillatory components (Figure S3B). We thus detected bursts within each gamma band and computed their main spectral components using broadband LFP spectrograms. This indicated a mean frequency of ~31 Hz for slow gamma in the radiatum, and ~35 Hz in the granular layer (Figure 2C; left). Mid gamma displayed a main frequency of ~68 Hz across all layers (Figure 2C; middle). Fast gamma exhibited a main frequency of ~130 Hz in the pyramidal layer, and ~120 Hz in the radiatum, molecular layer, and granular layer (Figure 2C; right).

Previous work showed that slow and mid gamma amplitude increases with running speed in mice^46,66^. Consistent with this, we observed that all rhythms increased in power with speed [speed modulation index, averaged across mice (99% CI): pyramidal fast gamma, 4.75 (3.58–6.03); radiatum slow gamma, 5.42 (4.19–6.58); radiatum fast gamma, 2.85 (1.74–4.04); lacunosum-moleculare mid gamma, 3.38 (1.95–4.94); mid-molecular fast gamma, 5.40 (4.65–6.11); granular fast gamma, 2.29 (1.64–3.08)].

In sum, signals recorded with linear probes consistently revealed slow gamma oscillations in the radiatum and granular layers, along with a widespread mid-gamma component across all CA1-to-DG layers (Figure 2D). This profiling also identified four distinct fast gamma components localized to the pyramidal, radiatum, mid-molecular, and granular layers. Lastly, the consistency of gamma profiles across mice provides robust cross-validation for hippocampal layer delineation, as gamma oscillations were not used to compute the LFP embedding.

### Laminar signatures retrieved in independently movable tetrode recordings

To evaluate the generalizability of hippocampal layer profiling across mice and recording techniques, we next used the embedding to guide tetrode placement in mice implanted with independently movable tetrodes. Each tetrode was lowered stepwise, with signals recorded at each position, while one tetrode remained in the pyramidal layer as a reference for ripple detection and theta phase.

**Figure 3:**
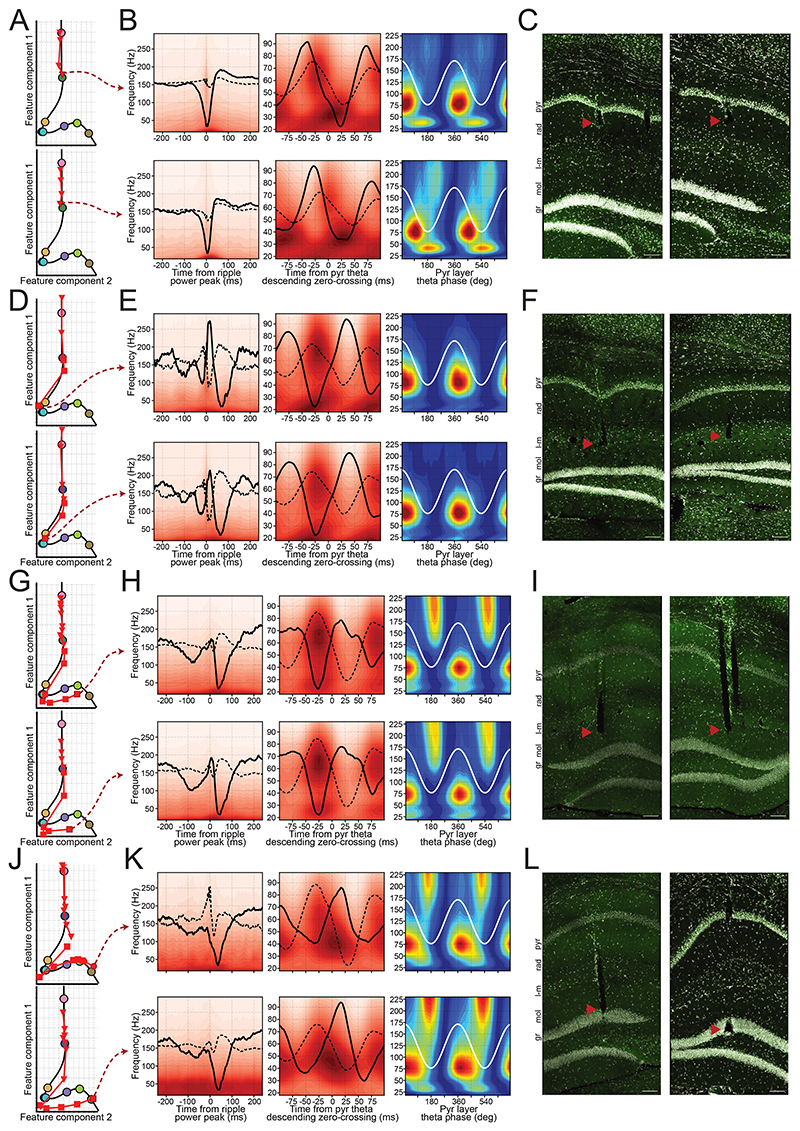
Validation of LFP profiles using tetrode placement in the feature embedding. (A-C) Tetrode placement in the radiatum layer. (A) Trajectories in feature space, with each triangle representing a tetrode's projection for a recording session (connecting lines indicate session sequence). Each panel shows an example tetrode. (B) Sharp-wave and theta-gamma profiles obtained from these tetrodes immediately before perfusion. Left: SWR waveform obtained from the corresponding tetrode (solid line) and from the pyramidal layer reference (dashed line); heatmap displays amplitude across frequencies. Middle and right panels: Theta-gamma profiles as shown in Figure 2. (C) Histological confirmation of tetrode placement. Red arrowheads indicate tetrodes tips, with layers visualized using DAPI staining (white) and an empty channel (green). Scale bars: 100 μm. (D-F) Same analysis for tetrodes targeting the lacunosum-moleculare layer. Each marker represents same-day sessions, with triangles and squares indicating consecutive days. (G-I) Same analysis for tetrodes targeting the DG molecular layer. (J-L) Same analysis for tetrodes targeting the DG granular layer. Circles indicate a third recording day.

The procedure began with tetrodes positioned in the pyramidal layer, where ripples were clearly visible and the tetrode’s embedding projection was close to the pyramidal layer coordinate. For tetrodes aimed at the radiatum, signals were recorded at each lowering step, and their projections were tracked until reaching the radiatum coordinate (Figure 3A). At that point, SWR and theta waveforms were consistent with those of radiatum channels in the silicon probe data (Figure 3B). Subsequent histological evaluation confirmed that radiatum-targeted tetrodes were positioned within the predicted layer (Figure 3C). The same procedure was applied to tetrodes targeting the lacunosum-moleculare, mid-molecular, and granular layers (Figure 3D–L). These results, combining tetrode recordings with histological verification, validate the embedding layer prediction.

The gamma signatures observed with embedding-guided, anatomically-validated tetrodes were consistent with those obtained in silicon probe recordings (Figures 3 and S4). Specifically, fast gamma oscillations were detected in the pyramidal, radiatum, and mid-molecular layers, just after the pyramidal theta trough, while in the granular layer they preceded the theta trough. Radiatum fast gamma was more prominent in the distal part of the layer, below its "center" coordinate (Figure S5). Slow gamma components were consistently present in the radiatum and granular layers, as observed with silicon probes. A beta component (~18–35 Hz) was detected in molecular layer channels, while mid gamma (~50–90 Hz) was observed across all layers, also consistent with silicon probe recordings.

### Spiking profiles of pyramidal layer neurons across gamma oscillations

**Figure 4:**
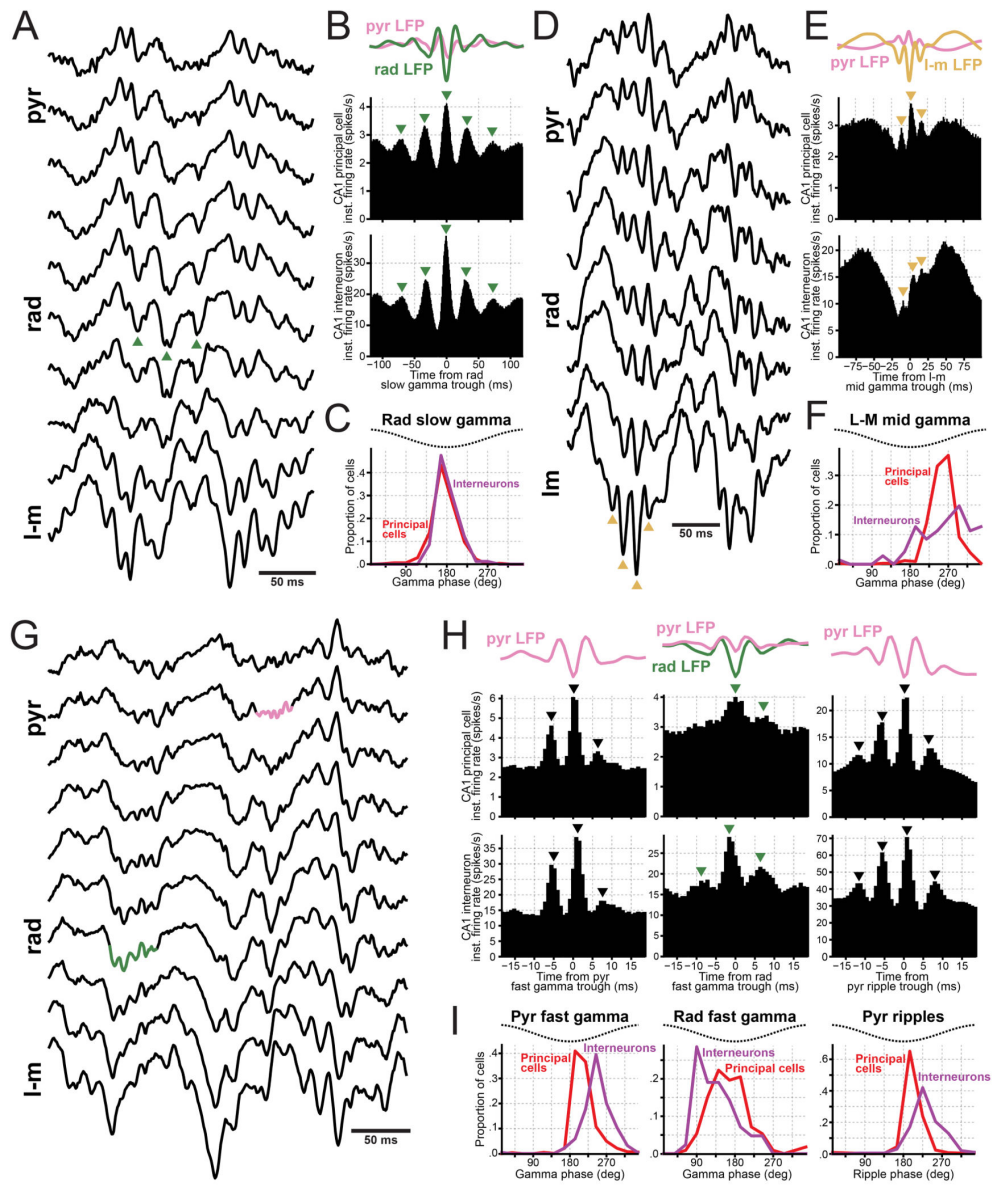
Spiking correlates of CA1 principal cells and interneurons to gamma rhythms. (A-C) Spike correlates of radiatum slow gamma. (A) Example silicon probe recordings showing radiatum slow gamma. Individual slow gamma troughs are marked by green triangles. (B) LFP averages triggered by slow gamma troughs (top panel), along with the averaged activity of principal cells (middle panel) and interneurons (bottom panel). Data are aggregated from 22 recording days across 10 mice. Within each recording session, the combined activity of all principal cells or all interneurons was aligned to a single slow gamma trough per theta cycle. Session averages were combined to produce the displayed grand average. (C) Average gamma phase histogram for principal cells and interneurons. Only cells significantly coupled to slow gamma (p<0.01) were included: 492 principal cells and 82 interneurons. Slow gamma mean firing phase: 176° for principal cells (99% CI: 173 – 179), and 179° for interneurons (99% CI: 172 – 185). Phase difference (principal – interneurons): -2.5° (99% CI: -9.7 – 4.2), bootstrap p = 0.345. (D-F) Same as in (A-C), but for lacunosum-moleculare mid gamma. (E) Same as in (B) using sessions with a lacunosum-moleculare tetrode (32 recording days from 13 mice). (F) Same as (C), but for mid gamma. Includes 333 principal cells and 70 interneurons significantly coupled to mid gamma. Mid gamma mean firing phase: 258° for principal cells (99% CI: 254 – 262), and 274° for interneurons (99% CI: 255 – 293). (G-I) Same as in (A-C), but for fast gamma oscillations. (G) Silicon probe recordings showing fast gamma oscillations. (H) Same analysis as in (B), but for pyramidal fast gamma, radiatum fast gamma and ripples. Pyramidal fast gamma and ripple data: 59 recording days (including both awake and sleep periods) from 20 mice. Radiatum fast gamma data: 17 recording days from 11 mice with a distal radiatum tetrode. (I) Same analysis as (C). Includes 789 principal cells and 220 interneurons significantly coupled to pyramidal fast gamma, and 112 principal cells and 42 interneurons significantly coupled to radiatum fast gamma. Pyramidal fast gamma mean firing phase: 221° for principal cells (99% CI: 219 – 224), and 243° for interneurons (99% CI: 238 – 248); phase difference (principal – interneurons): -21.8° (99% CI: -27.2 – -16.3), bootstrap p < 10^-5^. Radiatum fast gamma mean firing phase: 170° for principal cells (99% CI: 160 – 180), and 134° for interneurons (99% CI: 113 – 152); phase difference (principal – interneurons): 36.5° (99% CI: 13.9 – 57.5), bootstrap p = 8x10^-5^.

Gamma oscillations are thought to reflect postsynaptic potentials influencing local spiking activity^67^. Investigating the temporal relationship between neuronal spiking and gamma oscillations provides insights into their underlying mechanisms and functions. We observed a strong relationship between the gamma phase and the spike timing in both principal cells and pyramidal layer interneurons (Figure 4). Aligning the activity of these populations to a single trough of radiatum slow gamma per theta cycle revealed clear slow gamma-paced spiking, spanning at least five cycles (Figure 4A,B). The mean phase for both CA1 principal cells and pyramidal layer interneurons was consistent at the radiatum slow gamma troughs (Figure 4C). Applying the same analysis to mid gamma in the lacunosum-moleculare layer revealed three cycles of mid gamma-paced spiking in CA1 pyramidal layer cells (Figure 4D,E). While CA1 principal cells consistently fired preferentially around lacunosum-moleculare mid gamma ascending phase, pyramidal layer interneurons displayed varied firing phases from the mid gamma trough to its peak (Figure 4F).

We next analyzed spike modulation by fast gamma oscillations recorded either from the pyramidal layer or distal radiatum (Figure 4G-I). Gamma trough-triggered averages for each layer-specific fast gamma revealed 2 to 3 cycles of fast gamma-modulated spiking in CA1 principal cells and pyramidal layer interneurons (Figure 4H; left and middle columns). These findings support the existence of genuine fast gamma oscillations, beyond potential spike-leakage artifacts. For comparison, we repeated this analysis for ripples, which share a similar frequency range to fast gamma (Figure 4H; right). Consistent with previous work^20,68^, principal cells fired around the ripple trough, slightly preceding pyramidal layer interneurons (Figure 4I). Similarly, both cell types preferred the ascending phase of pyramidal fast gamma, with principal cells firing earlier (Figure 4I). In contrast, radiatum fast gamma exhibited the opposite relationship, with interneurons firing earlier than principal cells (Figure 4I; middle). These results suggest that pyramidal and radiatum fast gamma oscillations are generated by distinct mechanisms.

### Profiling CA1 principal cells from deep to superficial pyramidal sublayers

**Figure 5:**
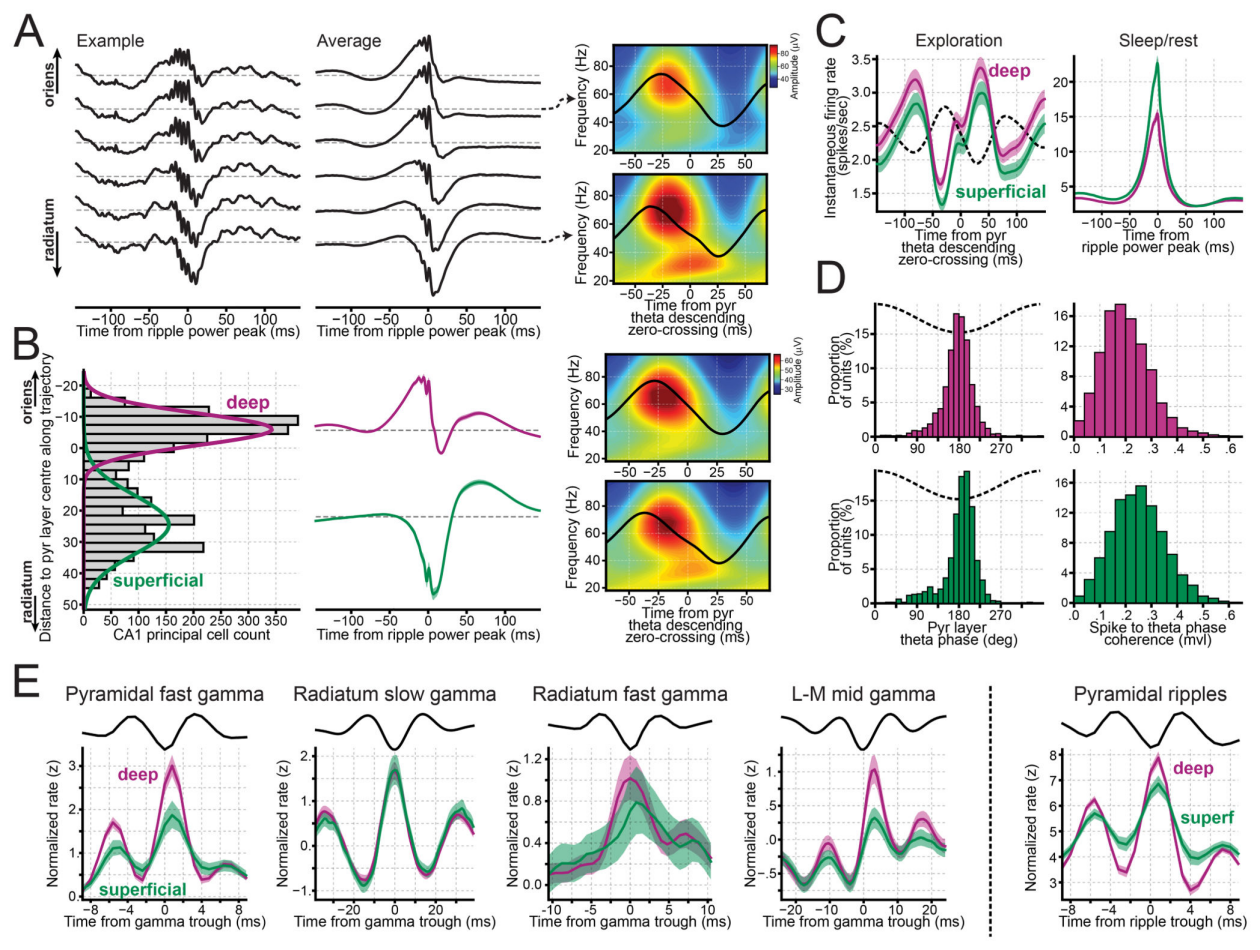
Firing behavior of CA1 principal cells in deep and superficial pyramidal sublayers. (A) Axial profile of SWR waveforms recorded with a silicon probe (20 μm contact spacing). Left: example SWR event. Middle: average SWR waveform across all events in the session. Right: theta-nested gamma profile and the local LFPs aligned to the descending zero-crossing of pyramidal theta, for the channels indicated by arrows. (B) Classification of principal cells as deep and superficial. Left: distribution of tetrodes projected onto the linearized trajectory (as in Figure 1F). Overlaid traces represent Gaussian components from a GMM fit. Middle: SWR waveforms from tetrodes assigned to each Gaussian component. Right: mean theta-nested gamma profiles from the same tetrodes. (C) Mean instantaneous firing rate of deep and superficial pyramidal cells during theta and SWRs. Left: activity aligned to the descending zero-crossing of pyramidal theta for both populations. Right: activity aligned to the ripple power peak. (D) Theta coupling of deep and superficial pyramidal cells. Left: histograms of the mean firing phase for both populations. Right: distribution of coupling strength for each population. (E) Z-scored firing rate of deep and superficial cells, aligned to the troughs of CA1 gamma oscillations and ripples (one trough per theta cycle). Lines above each panel display the average LFP from the corresponding layer. Solid lines indicate means, shaded areas indicate 99% bootstrap confidence intervals.

Recent studies have highlighted differences in the firing characteristics of CA1 principal cells along the radial axis of the pyramidal layer^53,54,69,70^. Using our embedding approach, we resolved subpopulations of (tetrode-recorded) principal cells. This sublayer resolution emerged primarily from the gradual progression of the sharp-wave waveform—detected as a positive deflection near the oriens layer, shifting to a minor negative deflection within the pyramidal layer, and ultimately becoming the distinct negative sharp-wave in radiatum (Figure 5A). Projecting signals from individual tetrodes yielded a bimodal distribution of embedding coordinates (Figure 5B; left). This bimodality reflected how minor adjustments in tetrode position around the sharp-wave polarity inversion produced marked shifts in the embedding projection, resulting in a gap in the projection distribution.

Consistent with the silicon probe recordings (Figure 5A), tetrodes positioned in the pyramidal layer closer to radiatum exhibited a clearer theta-nested slow gamma component than those near the oriens (Figure 5B; right panels). We found a significant relationship between sharp-wave amplitude and slow gamma power across tetrodes (Figure S6A; Spearman correlation = 0.53, p < 10^-10^). No such correlation was observed with mid gamma power (Spearman correlation = 0.027, p = 0.46).

Next, we categorized CA1 pyramidal cells into deep and superficial populations using a 2-component Gaussian Mixture Model classifier applied to their linearized coordinates (Figure 5B). During awake theta oscillations, deep pyramidal cells fired at a higher rate than superficial cells [Figure 5C; mean rate (99% CI): deep, 2.56 (2.43 – 2.69) spikes/sec; superficial, 2.24 (2.11 – 2.37) spikes/sec; p < 10^-5^ for both bootstrap and permutation tests]. In contrast, during sleep/rest SWRs (analyzed using a 10-ms window centered at the ripple power peak), superficial cells fired more than deep cells [Figure 5C; mean rate (99% CI): superficial, 19.90 (18.96 – 20.88) spikes/sec; deep, 13.62 (13.13 – 14.14) spikes/sec; p < 10^-5^ for both bootstrap and permutation tests].

The mean theta firing phases also differed between the pyramidal sublayer groups. Deep principal cells fired with an earlier mean theta phase than superficial cells (Figure 5D and Table S2; p < 10^-5^ for both bootstrap and permutation tests). Superficial cells exhibited stronger spike-to-theta phase coherence (Figure 5D and Table S2; p < 10^-5^ for both bootstrap and permutation tests). This difference in coherence stemmed from deep pyramidal cells firing proportionally more spikes around the theta peak (Figure 5C), nudging their mean phase closer to the peak and resulting in reduced modulation depth.

Given that low and high firing rate CA1 principal cells are hypothesized to exhibit different properties ^71,72^, it remained plausible that the observed disparities in mean theta phase and coherence arose from firing rate differences rather than anatomical position. To address this, we conducted an additional analysis, subsampling deep and superficial cells to match their firing rate distributions (Figure S6B). After matching firing rates, superficial cells still exhibited a later mean theta phase than deep cells [mean phase difference (99% CI): 7.42° (6.26 – 8.56), p < 10^-5^]. Similarly, superficial cells remained more strongly coupled to the theta phase [mean vector length difference (99% CI): 0.029 (0.026 – 0.033), p < 10^-5^].

We further examined the coupling levels between gamma oscillations and pyramidal cell groups (Figure 5E and Table S2). While slow gamma coupling was similar in both groups, deep cells exhibited stronger coupling to pyramidal and radiatum fast gammas, mid gamma, and ripple oscillations (Table S2). These findings highlight distinct firing properties along the radial axis of the CA1 pyramidal layer, underscoring the importance of considering this sublayer heterogeneity.

### CA1 interneuron firing behavior follows a laminar organization

**Figure 6:**
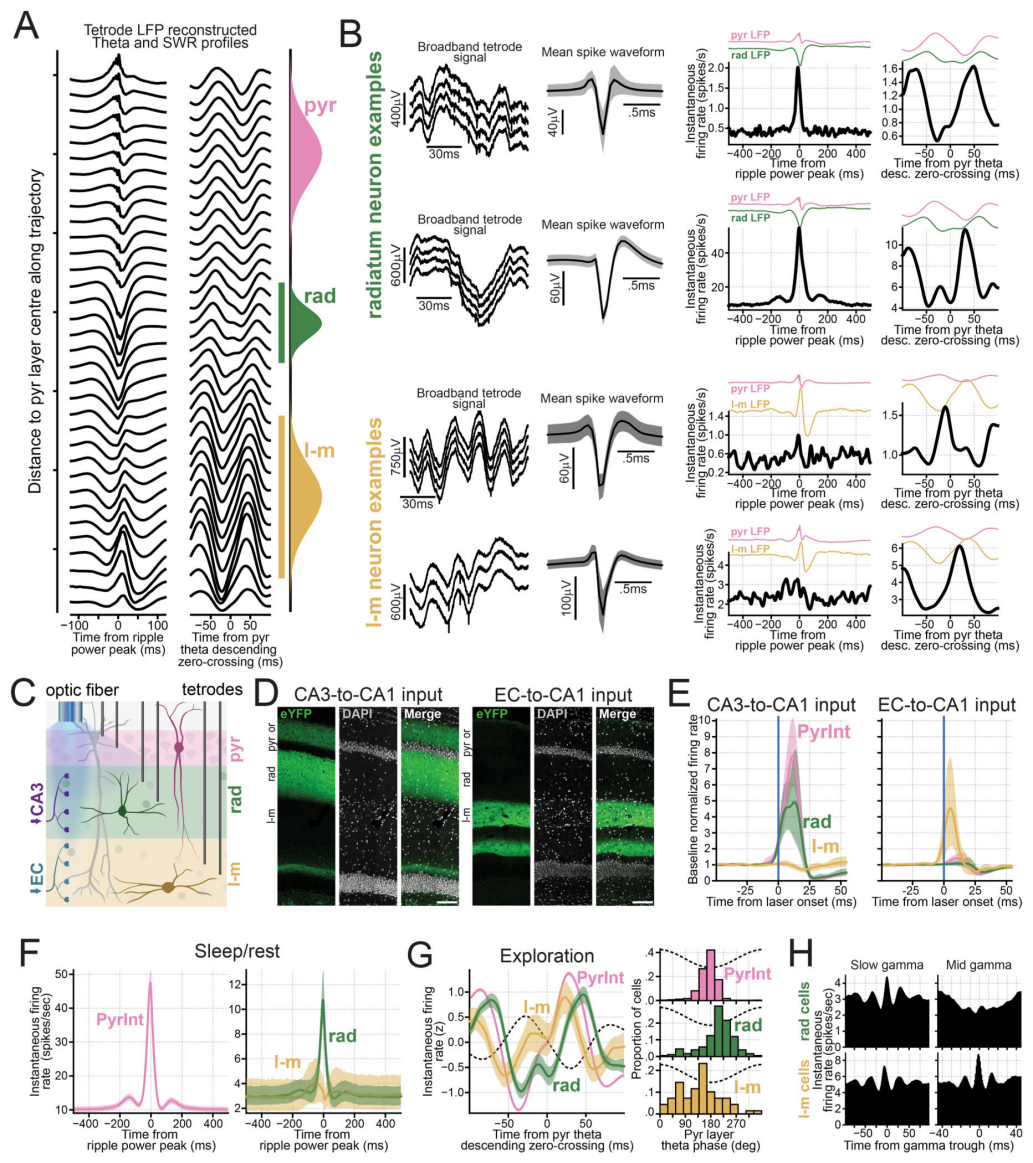
Firing behavior of CA1 interneurons in radiatum and lacunosum-moleculare layers. (A) CA1 laminar profile reconstructed from tetrode recordings. Sharp-wave and theta waveforms were recorded using independently movable tetrodes. Average waveforms from tetrodes at varying distances from the pyramidal layer are shown. Gaussian fits (from Figure 1F) are displayed on the right (solid lines denote the 99% fit areas). Units recorded within these ranges were classified as radiatum (rad) or lacunosum-moleculare (l-m) interneurons. (B) Example rad and l-m neurons. Left: broadband signals where neurons were detected. Adjacent panels show mean spike waveforms (shading denotes interquartile range). Right: SWR response and theta phase modulation for each neuron. (C-E) Rad and l-m interneurons respond to distinct upstream inputs. (C) Optic fibers and tetrodes were implanted in CA1 to monitor neuronal activity during optogenetic stimulation of CA3 or EC terminals (see Methods). (D) CA3→CA1 inputs were targeted by transducing CA3 cells with a Cre-dependent channelrhodopsin-2 (ChR2)-YFP vector in Grik4-Cre mice; EC→CA1 inputs were targeted by transducing EC cells with a CamKII-driven ChR2-YFP construct in wild-type mice (Figure S7C). Images show ChR2-YFP-labeled terminals in CA1 from CA3 (left) and EC (right). Scale bars, 100 μm. (E) Optogenetic stimulation of CA3→CA1 inputs activated 64.5% of pyramidal layer interneurons (PyrInt; 40/62) and 34.8% of rad interneurons (23/66), but no l-m cells (0/18). EC→CA1 input stimulation recruited 42.3% of l-m cells (11/26), with minimal activation of PyrInt (2/46) and no rad neuron responses (0/63). Significant responses were defined as firing rates exceeding the baseline mean by 2 standard deviations and independently confirmed with a p < 0.01 threshold in a permutation test. (F) Mean firing rate of PyrInt, rad and l-m interneurons during SWRs. (G) Theta modulation during exploration. Left: z-scored firing rates aligned to the descending zero-crossing of pyramidal theta. Right: distribution of mean theta phases. (H) Gamma modulation of rad and l-m neurons (as in Figure 4B,E). Shaded areas in panels E, F, and G denote 99% bootstrap confidence intervals across cells.

CA1 layers host highly diverse arrays of interneurons^10,55,73,74^. Unlike the pyramidal layer, which contains the highest density of somata, the radiatum and lacunosum-moleculare layers have sparse interneuron populations. This anatomical sparsity imposes a significant challenge for *in vivo* electrophysiological recordings, contributing to our limited understanding of their spiking dynamics. Using our embedding approach, we classified interneurons in our tetrode dataset by their somatic layer (Figure 6A). In addition to 389 interneurons recorded in the pyramidal layer, we identified 176 and 69 interneurons in the radiatum and lacunosum-moleculare layers, respectively (Figure 6B). By optogenetically targeting CA3 versus EC inputs using Channelrhodosin-2 in some of these mice, we confirmed that the interneurons recorded in these layers receive distinct, radially organized upstream inputs (Figure 6C-E)^75^. Specifically, optogenetic stimulation of CA3 terminals strongly entrained interneurons in radiatum (and stratum pyramidale) with minimal effect on lacunosum-moleculare cells; while EC terminal stimulation strongly entrained lacunosum-moleculare interneurons with little effect on other layer interneurons (Figure 6E).

During sleep/rest periods outside of SWRs, pyramidal interneurons were the most active [mean rate (99% CI): 10.3 (9.5–11.1) spikes/sec], while radiatum and lacunosum-moleculare interneurons exhibited similarly lower activity [mean rate (99% CI): radiatum, 2.9 (2.3–3.8) spikes/sec; lacunosum-moleculare, 3.0 (1.9–4.5) spikes/sec]. During SWRs, pyramidal and radiatum interneurons showed strong transient increase in firing, whereas lacunosum-moleculare cells showed minimal to non-significant responses (Figure 6F). During awake theta, pyramidal interneurons were the most active [mean rate (99% CI): 14.4 (13.3–15.5) spikes/sec], followed by lacunosum-moleculare [5.5 (3.2–8.4) spikes/sec] and radiatum cells [3.1 (2.2–4.4) spikes/sec]. Interneurons in different layers fired with different preferred theta phases (Figure 6G): lacunosum-moleculare cells fired earliest [mean phase (99% CI): 133° (109–157)], followed by pyramidal [172° (170–174)] and radiatum [214° (205–222)] interneurons. Thus, CA1 interneurons and principal cells exhibit a layer-selective activity profile (Figure S7A).

In terms of theta-nested gamma oscillations, radiatum and lacunosum-moleculare interneurons were weakly modulated by fast gamma from both pyramidal and radiatum layers (Figure S7B). Radiatum slow gamma oscillations strongly modulated interneurons across all CA1 layers (Figures 4E,6H and S7C). Lacunosum-moleculare interneurons showed strong coupling to lacunosum-moleculare mid gamma, whereas radiatum interneurons (like pyramidal layer interneurons; Figure 4E), were only weakly modulated by this oscillation (Figures 6H and S7C).

## Discussion

### Mapping hippocampal layers through electrophysiological signatures

The hippocampus comprises a complex network of interrelated microcircuits. Understanding their operations is key to revealing how the hippocampus supports diverse behaviors. By placing electrodes in different parts of this circuitry, we capture a myriad of electrophysiological patterns, which reflect both the activation of individual circuit components and their dynamic interactions. Interpreting these patterns provides insights into the cellular mechanisms of each microcircuit and their functions.

We developed an embedding that maps hippocampal layers based on theta and sharp-wave waveforms. Channels from the same layer consistently clustered together across mice, and the embedding reliably guided layer targeting in movable tetrode recordings. These results indicate that mapping layers using electrophysiological features, rather than absolute anatomical depth, can homogenize laminar identification across variations in penetration angle, anatomical scaling, and experimental setup.

Of note, positions in the embedding space do not map linearly onto absolute anatomical depth. Instead, the feature space reflects how rapidly electrophysiological features change across layers. As a result, regions with minimal changes — such as the transition from lacunosum-moleculare to the outer molecular layer — are compressed, whereas regions with sharp transitions— such as from deep to superficial CA1, where sharp-wave polarity reverses — are expanded. This nonlinearity explains the apparent bimodality in tetrode embedding projections (Figure 5B). Thus, although the CA1 pyramidal layer is anatomically thinner than the combined lacunosum-moleculare and outer molecular layers, it occupies a substantially larger portion of the embedding space.

We propose that this approach is broadly adaptable to other layered brain regions and across species. Examining the phase reversal of delta waves and spindles in the neocortex^76–79^ could reveal nuanced laminar patterns. Extending such analyses across neocortical areas — from primary to associative — or comparing these networks across species could offer deeper insights into circuit organization and function. Such efforts could also align with and expand on studies suggesting canonical, layer-based mechanisms for cortical computation^80,81^.

### Limitations of the study

Our embedding did not differentiate between lacunosum-moleculare and outer molecular layers, reflecting the highly similar SWR and theta waveforms. We also found no consistent differences in their theta-gamma profiles, and their CSD signals were highly correlated during both SWR and theta (Figures 1C and S1), suggesting that the underlying synaptic currents related to these events are largely indistinguishable. One shared input to these layers, but not to the mid molecular layer, is the lateral entorhinal cortex (LEC)^82^. Although both LEC and the medial entorhinal cortex (MEC) project to lacunosum-moleculare, the organization of their axonal projections along the septo-temporal and proximal-distal axes of CA1^82–84^ may bias lacunosum-moleculare signals toward a LEC-dominated pattern, thereby approximating it to outer molecular layer electrophysiologically at our recording sites. Additionally, certain interneuron types targeting lacunosum-moleculare in dorsal CA1 extend axonal branches into the DG molecular layer, potentially contributing to the shared patterns observed across these regions^85,86^. Although our focus here was on the radial axis of the dorsal hippocampus, these observations highlight the importance of examining how electrophysiological patterns vary along anatomical axes, and how such variations correlate to known gradients of extrinsic and local inputs.

Our framework relies on CSD-based layer definitions to create reference points for aligning feature trajectories across animals. In its current form, applying this method without a comparable ground truth would require adapting the alignment strategy. Moreover, we fit the embedding using probes with inter-channel spacing matched to the anatomical spacing of the layers studied. When channels are under-sampled (reducing spatial resolution), crucial information needed to separate layers is lost (Figure S2C). Thus, applying this framework to other regions requires a comparable dataset with simultaneous recordings that sample a spatial axis at sufficient spatial resolution to resolve the relevant layers.

**Figure 7:**
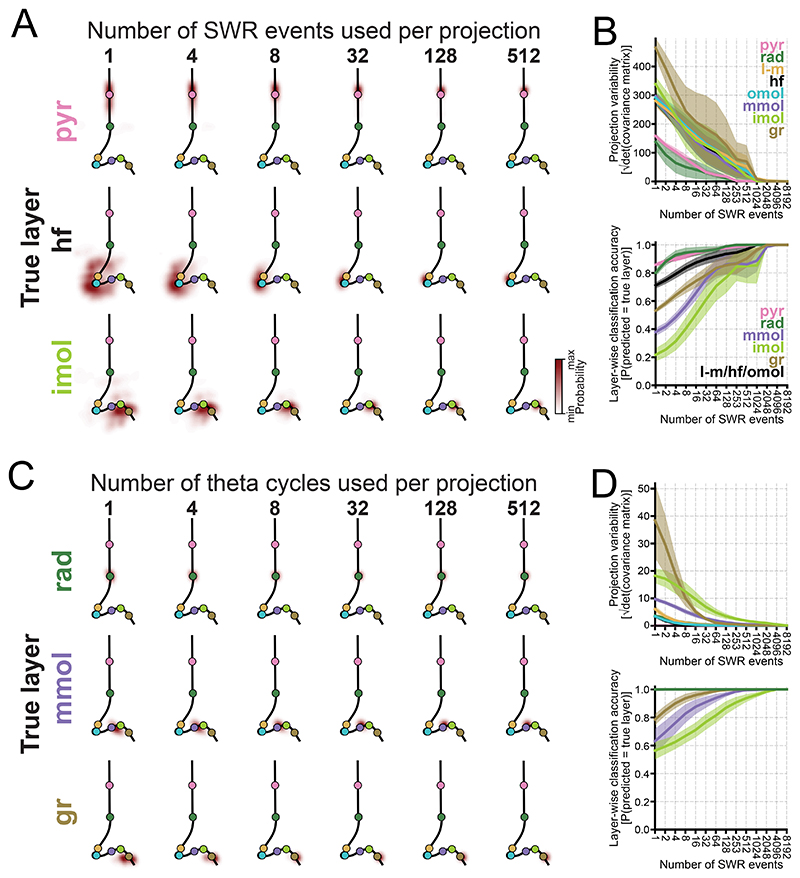
Effect of event sample size on projection variability and classification performance. (A) We evaluated how the number of SWR events used affects the embedding projection and layer classification. All available theta cycles were used while varying the number of randomly sampled SWR events. For representative channels from the pyramidal layer, hippocampal fissure (hf), and inner molecular layer (imol), projection histograms are shown for different SWR sample sizes. Smaller sample sizes yield broader distributions, indicating that a few hundred SWR events are necessary for convergence to the average projection coordinate. (B) Quantification of projection variability and classification accuracy across mice and layers. Classification accuracy is the proportion of projections correctly assigned to the ground-truth layer using a classifier (as in Figure 1H). (C) Same as in (A) but using all available SWR events while varying the number of theta cycles. Shown are representative channels from the radiatum, mid molecular (mmol), and granular (gr) layers. (D) Same as in (B) but for theta cycle subsampling.

As both SWR^87–90^ and theta^91,92^ waveforms exhibit variability, averaging across multiple events is necessary to obtain stable mean waveforms for mapping channels into the embedding. To estimate how many SWR events and theta cycles are needed within our framework, we randomly sampled varying numbers of events for each pattern and evaluated how sample size affected their projections. When using all available theta cycles and subsampling SWR events, we observed a substantial variability in the projection of the same channel (Figure 7A,B). Strikingly, when repeating the same analysis for theta cycles (using all available SWR events), the projection variability was substantially lower (Figure 7C,D). This shows that the theta waveform features that fluctuate across cycles are largely orthogonal to the embedding. Thus, resolving a layer such that it can be distinguished from others in the embedding typically requires hundreds of SWR events, whereas far fewer theta cycles suffice.

### On SWR diversity and embedding projection variability

Recent studies have shown that LFP signals recorded from a single site can reflect underlying currents across layers. Sebastián et al.^93^ used topological analysis of SWR waveforms recorded at the pyramidal layer to reveal a low-dimensional structure mapping onto CSD profiles. Castelli et al.^90^ showed that ripple waveform at stratum pyramidale predicts SWR CSD profiles. Similarly, pyramidal layer LFPs can be used to distinguish between DS types classically defined by CSD analysis^14,94^. While our framework is conceptually aligned with these studies in retrieving CSD information from single-site LFPs, we have not here explored SWR diversity. However, when projecting a single SWR event onto the embedding, coordinates showed substantial spread around its ‘converging’ position (Figure 7). Although this may reflect an under-sampling issue—where more events must be averaged to reduce ‘noise’—it is possible that this variability reflects meaningful physiological processes. For instance, one could test whether CA2 activity preceding CA1 SWRs^95^ biases embedding projections.

### Network layering and cellular basis of theta-nested gamma oscillations

CA1 slow gamma is thought to originate in the radiatum^41,42,46,65^, a layer receiving CA3 projections^83,96^. CA3 LFPs prominently express slow gamma^42,43^, and CA1-CA3 coherence is strong within this frequency band^39,42,97^. Suppressing CA3 terminals optogenetically reduces slow gamma power without affecting mid gamma^98^. Activating CA3 parvalbumin-expressing interneurons, which suppresses CA3 principal cell output, also reduces CA1 slow gamma^99,100^. Here, slow gamma oscillations were prominent in the CA1 radiatum. The slow gamma observed in the pyramidal sublayer adjacent to radiatum is presumably volume-conducted from radiatum. The dense cellular packing of the pyramidal layer likely acts as a barrier, limiting propagation of this signal toward stratum oriens. In lacunosum-moleculare, slow gamma was overshadowed by more dominant mid gamma oscillations.

Mid gamma oscillations are proposed to originate from the lacunosum-moleculare^41,42,46^, the main CA1 target of EC layer 3 inputs^82,101^. Optogenetically disturbing EC output diminishes the mid gamma theta-phase modulation, while leaving radiatum slow gamma unaffected^102^. Intriguingly, we detected mid gamma activity in more distant layers like the oriens. While volume conduction may explain this, radiatum slow gamma does not similarly cross the pyramidal layer. Moreover, despite low-pass filtering of apical dendrite currents as they travel to the soma, CA1 principal cell spiking remained clearly modulated by mid gamma. This probably arises from EC-driven mid gamma inputs entraining lacunosum-moleculare interneurons^6^ (Figure 6E), which in turn synchronize pyramidal cell firing with mid gamma. However, some interneurons act differently; for instance, neurogliaform cells in lacunosum-moleculare can decouple mid gamma from CA1 pyramidal cell firing^103^.

The weak mid gamma modulation of pyramidal layer interneurons—contrasting with the strong modulation observed in lacunosum-moleculare interneurons—suggests that the mid gamma network operates in parallel to the fast gamma circuitry. Its distributed presence across all layers indicates a broader integrative role, potentially coordinating inputs beyond EC, including CA2 projections to the stratum oriens^104^. Thus, although mid gamma may be mainly driven by EC, it is not simply transmitted feedforward but emerges from a local CA1 circuit involving lacunosum-moleculare interneurons.

CA1 fast gamma oscillations generated within the pyramidal layer likely reflect interactions between principal cells and pyramidal layer interneurons^6,41^. Although spike contamination is a valid concern^65,105^, our previous^46^ and current observations demonstrate genuine rhythmicity. We propose that pyramidal fast gamma and ripples arise from a common cellular substrate, differing only in their initiating drive: fast gamma is evoked by theta-related inputs, while ripples are driven by sharp-wave-related inputs.

Interestingly, the timing relationship between pyramidal cells and interneurons associated with the radiatum fast gamma cannot be explained by the same mechanism. Instead, this rhythm likely stems from feedforward inputs targeting the radiatum and/or lacunosum-moleculare layers. Fast gamma oscillations in DG have been documented previously^106–108^ and shown to be distinct from CA1 fast gamma^107^. Here, we extend these findings by distinguishing two DG fast gamma rhythms: one more pronounced in the molecular layer, the other in the granular layer.

Slow gamma oscillations have been identified in DG^106,107^. Given their theta phase alignment with CA1 radiatum slow gamma and the pronounced entrainment of DG cells by CA1 slow gamma (even stronger than in CA1 cells), both rhythms may share a common source^107^. One possibility is that they originate in DG, propagate to CA3, and subsequently manifest in CA1 radiatum^38,107,109^. Alternatively, the source may be extra-hippocampal, possibly within the medial septum^110^. Moreover, Fernandez-Ruiz et al^106^ suggested that DG slow gamma arises from LEC inputs.

We further identified a beta component in the DG molecular layer (Figures 2,3 and S2A,S3,S4), occasionally observed in lacunosum-moleculare (Figure S5). This component shares a similar frequency range and theta-phase modulation with a beta component previously described in the CA1 pyramidal layer using a method designed to detect transient signals not apparent in averaging analyses^46^. An oscillation in a similar frequency, termed ‘beta2’, has also been reported in the hippocampus^111,112^. Beta2 partly disrupt ongoing theta and persists for several cycles^111^, rather than being modulated by theta phase. While the theta-phase-modulated beta has been associated with memory retrieval^46^, beta2 has been linked to novelty detection^111–113^. Additionally, 4-Hz-paced activity in ventral tegmental area coordinates beta activity in distributed brain regions, including the hippocampus^114^. It remains unclear whether these beta-band rhythms reflect a shared cellular mechanism recruited in different behavioral states or arise from distinct oscillators with overlapping frequency bands.

### Radial organization of principal cell firing behavior within the CA1 pyramidal layer

Cells in the deep CA1 pyramidal sublayer are more active during awake theta^69^, whereas superficial cells are more active in sleep SWRs^68^ (Figure 5C). This SWR-related rate difference aligns with *in vivo* patch-clamp studies showing that superficial cells receive heightened excitatory drive during SWRs^88,115^. Superficial pyramidal cells exhibited stronger theta-phase coupling than deep cells, while deep cells exhibited stronger synchrony with ripples and gamma oscillations (except slow gamma). The enhanced gamma coupling in deep cells likely reflects the stronger local fast-spiking interneuron control over deep cells^116^. The enhanced response of superficial cells to CA3 inputs^115^ may explain their similar modulation by slow gamma. Deep cells may be more responsive to gamma-associated currents, leaving their activity less dominated by theta and resulting in proportionally more spikes outside their preferred theta phase. The distinction between deep and superficial CA1 pyramidal cells is emerging as a critical factor in hippocampal processing^53^. These results emphasize that hippocampal functions cannot be fully understood without accounting for CA1 pyramidal sublayer-specific differences.

### Radial organization of interneuron firing behavior across CA1 layers

We recorded single-neuron activity from the sparse CA1 radiatum and lacunosum-moleculare layers. Traditionally, recordings in freely-moving animals have targeted the pyramidal layer, using its high neuron density and well-characterized electrophysiological markers (e.g., ripples). Comparable readouts have been lacking for the more superficial layers. Our LFP embedding enables precise targeting of radiatum and lacunosum-moleculare neurons.

We found significant firing differences across CA1 layers. Radiatum interneurons were strongly recruited by CA3 terminal stimulation and during SWRs, which are primarily driven by CA3 inputs^21^. In contrast, lacunosum-moleculare cells were recruited by EC terminal stimulation and showed the strongest coupling to mid-gamma oscillations, consistent with the association of this rhythm with EC inputs^39,40^. These populations also differed in theta phase preference: lacunosum-moleculare neurons fired mostly just after the pyramidal theta peak; radiatum neurons fired preferentially just after the trough, consistent with EC and CA3 activity^7^.

Hippocampal interneurons exhibit heterogeneous firing properties associated with distinct morphological features^10,55,73^, which are not uniformly distributed along the CA1 radial axis^117^. Thus, while our results highlight a laminar organization of firing behavior, interneuron diversity remains an important factor. Consistent with previous work^9^, our results show that interneurons within the same layer receive common inputs, suggesting that somatic layer relates to activity dynamics. Fully testing this would require direct cell-type identification beyond somatic location, such as optogenetic tagging and/or intersectional (input- and molecularly-defined) approaches. Determining whether interneurons of the same type behave similarly across layers would help disentangle laminar and cell-type-specific influences and clarify how afferent connectivity shapes interneuron activity beyond intrinsic properties.

### On the significance of neural oscillations

LFP signals predominantly reflect synaptic activity^118^. For example, in the SWR complex, the radiatum sharp-wave reflects synchronized excitatory postsynaptic potentials from CA3 arriving via Schaffer collaterals^18,21,119^. Excitatory or inhibitory post-synaptic potentials occurring at a regular pace manifest in LFP signals as oscillations (e.g., gamma^67^). Different gamma oscillations are distinguished by the circuit mechanisms driving their rhythmic postsynaptic potentials. We identified four fast gamma oscillations with overlapping frequency bands, each manifesting in distinct layers of the CA1-DG axis. Particularly, the two fast gamma oscillations in CA1 — one in the pyramidal layer and the other in distal radiatum — differ in the timing relationships between pyramidal cells and interneurons. We also observed a beta component, with overlapping band with slow gamma. Past studies have defined slow gamma as the oscillation associated with CA3-to-CA1 inputs, most prominent during the descending phase of theta and linked to the CA1 radiatum^38,39,41,42,45,46^. In contrast, the beta component arises near the theta peak and was clearer in the DG molecular layers. The differences between beta and slow gamma highlight the need to define oscillations by their functional and anatomical substrates rather than by frequency alone. In line with this rationale, our study supports recent calls to revise the nomenclature of gamma oscillations to better reflect brain network physiology^40^. For example, hippocampal CA1 "slow gamma" would be more aptly termed "CA3-to-CA1 gamma", emphasizing its anatomical and functional basis over its frequency band. Similarly, CA1 pyramidal layer “fast gamma” would be more appropriately named CA1 “perisomatic gamma”^6^. Under this framework, a given (e.g., gamma) oscillation in another species would be considered equivalent if it shares the same cellular basis, even if it has a different frequency. Conversely, a signal with an identical frequency but recorded in different regions (e.g., hippocampus and visual cortex), would not be regarded as equivalent. Adopting this circuit-based approach would reduce confusion while emphasizing the physiological significance of brain rhythms.

## Resource Availability

### Lead contact

Further information and requests for resources and reagents should be directed to and will be fulfilled by the lead contact, David Dupret (david.dupret@bndu.ox.ac.uk).

### Materials availability

This study did not generate new unique reagents

## Star⋆Methods

### Key Resources Table

**Table:** 

REAGENT OR RESOURCE	SOURCE	IDENTIFIER
Bacterial and virus strains		
AAV2-EF1a-DIO-hChR2(H134R)- eYFP-WPRE	UNC Vector Core	Cat #AV3626
AAV9-CAMKII-hChR2(H134R)-eYFP-WPRE-hGH	Addgene	Cat #26969
Experimental models: Organisms/strains		
C57BL/6J mice	Charles River	IMSR_JAX:000664
Ndnf-IRES2-dgCre-D B6.Cg-Ndnf<tm1.1(folA/cre)Hze>/J	Jackson Laboratory	IMSR_JAX:028536
Vip-IRES-cre Vip<tm1(cre)Zjh>/J	Jackson Laboratory	IMSR_JAX:010908
G32-4 Cre C57BL/6-Tg(Grik4-cre)G32-4Stl/J	Jackson Laboratory	IMSR_JAX:006474
Sst-IRES-Cre Sst<tm2.1(cre)Zjh>/J	Jackson Laboratory	IMSR_JAX:013044
PV-Cre B6;129P2-Pvalb<tm1(cre)Arbr>/J	Jackson Laboratory	IMSR_JAX:008069
Software and algorithms		
Intan RHD2000	Intan Technologies	RHD2164
Positrack	Kevin Allen	n/a
Empirical Mode Decomposition in Python	Quinn et al.^120^	https://pypi.org/project/emd/
Hippocampal LFP embedding (Hipp-LFP-embedding)	Lopes-dos-Santos^121^, this study	concept https://doi.org/10.5281/zenodo.15275527
Kilosort via SpikeForest	Magland et al.^122^, Pachitariu et al.^123^	n/a
Other		
12pm tungsten wires	California Fine Wire	M294520
Silicon probe	NeuroNexus	A1x32-6mm-50-177- H32_21mm
Silicon probe	NeuroNexus	A1x32-5mm-25-177- H32_21mm
Silicon probe	NeuroNexus	A1x64-edge-6mm-20-177- H64LP_30mm
Silicon probe	Cambridge NeuroTech	ASSY-236 H3 Chronic 64- Molex
Optic fibers	Doric lenses	MFC_200/230- 0.37_10mm_RM3_FLT
Head-stage amplifier	Intan Technologies	RHD2164
473nm diode-pumped solid-state laser	Laser 2000	CL473-100

### Experimental Model and Study Participant Details

#### Animals

These experiments used 41 adult mice (4–6 months old; see Table S1). Animals were housed with their littermates up until the start of the experiment. All mice held in IVCs, with wooden chew stick, nestlets and free access to water and food *ad libitum* in a dedicated housing facility with a 12/12 h light/dark cycle (lights on at 07:00), 19–23°C ambient temperature and 40–70% humidity. Experimental procedures performed on mice in accordance with the Animals (Scientific Procedures) Act, 1986 (United Kingdom), with final ethical review and approval by the Science Regulation Unit of the UK Home Office for the animal studies.

### Method Details

#### Surgical procedure

All surgical procedures were performed under deep anesthesia using isoflurane (0.5–2%) and oxygen (2 l/min), with analgesia provided before (0.1 mg/kg vetergesic subcutaneous) and after (5 mg/kg Rimadyl (carpofen) subcutaneous) surgery.

For silicon probe recordings, mice were implanted with a single-shank silicon probe (Table S1) under stereotaxic control in reference to bregma, using central coordinates -2.0 mm anteroposterior from bregma, +1.7 mm lateral from bregma, and an initial depth of 1.5 mm ventral from the brain surface to span the somato-dendritic axis of CA1 principal cells and reach the DG. Following the implantation, the exposed parts of the silicon probe were covered with Vaseline® Healing Jelly, after which its plastic drive was secured to the skull using dental cement and stainless-steel anchor screws inserted into the skull. Two of the anchor screws, both above the cerebellum, were attached to a 50 μm tungsten wire (California Fine Wire) and served as ground. For the recordings, the silicon probe was positioned along the CA1-to-DG axis, using the rotations applied to its holding screw.

For tetrode recordings, mice were similarly implanted with a single microdrive containing 14 independently movable tetrodes, targeting the dorsal CA1 hippocampus. Tetrodes were constructed by twisting together four insulated tungsten wires (12 μm diameter, California Fine Wire) which were briefly heated to bind them together into a single bundle. Each tetrode was loaded in one cannula attached to a 6 mm long M1.0 screw to enable its independent manipulation of depth. To manipulate CA3 versus EC inputs in CA1 (Figure 6), mice were also implanted with a microdrive containing 14 independently movable tetrodes bilaterally targeting CA1, but further combined with two optic fibers (Doric Lenses Inc., Quebec, Canada) positioned bilaterally above CA1. Each drive was implanted under stereotaxic control in reference to bregma using the following coordinates. For pyramidal layer channels, the span was between AP -1.4 to -2.7 mm and ML 0.9 to 2.4 mm (bilateral). Tetrodes that delved deeper than the pyramidal layer were positioned within the range of AP –1.9 to –2.5 mm and ML 0.9 to 1.7 mm (bilateral). The initial depth of the tetrodes during the implantation surgery was 1.0 mm ventral from the brain surface. The minimal distance between neighboring tetrodes was 200 μm. Following the implantation, the exposed parts of the tetrodes were covered with paraffin wax, after which the drive was secured to the skull using dental cement and stainless-steel anchor screws inserted into the skull. Two of the anchor screws, both above the cerebellum, were attached to a 50 μm tungsten wire (California Fine Wire) and served as ground.

#### Viral injections

Mice were injected with one optogenetic construct prior to the microdrive implantation surgery, using the surgical procedure described above. Grik4-Cre heterozygote mice were injected in the CA3 region with a Cre-dependent channelrhodopsin-2 (ChR2) construct under the control of the EF1α promoter (UNC Cat #AV3626, AAV2-EF1a-DIO-hChR2(H134R)- eYFP-WPRE, 250nL, 2.5x10e11; Figure S7D). Wildtype mice were injected in the entorhinal cortex (EC) with a ChR2 construct under the control of the CAMKII promoter (Addgene Cat# 26969, AAV9-CAMKII-hChR2(H134R)-eYFP-WPRE-hGH, 250nL, 4.5x10e11; Figure S7D). Viral injections were performed using a glass micropipette (tip opening ~15–20 μm), which was slowly advanced into the brain (~10–20 μm/s) to the following coordinates (relative to bregma for AP/ML and brain surface for DV): CA3: AP -2.0 mm, ML -2.3 mm, DV -1.8 mm; EC: AP -4.85 mm, ML -3.45 mm, DV -1.5 mm. Upon reaching the target site, the pipette was held in position for at least 1 minute before initiating viral infusion at a rate of 0.1 μL/min. After delivery, the pipette was left in place for at least 5 minutes to minimize viral backflow, and then slowly retracted (~10–20 μm/s). The surgical site was closed using 6-0 Ethicon Vicryl sutures, and mice were allowed to recover for at least one week before further procedures.

#### Recording procedure

Following full recovery from the surgery, each mouse was first handled in a dedicated handling cloth, connected to the recording system, and exposed to an open-field enclosure to be familiarized with the recording procedure over a period of one week prior to the start of the experiment itself. During this period, animals were habituated to a sleep box (outer dimension: 12 cm width; 16 cm height) containing bedding from their home cage. This sleep box served all sleep recordings. When conducting awake sessions, the mice were set in open-field enclosures that varied in shape and had maximum side dimensions of 46 cm. For recordings using silicon probes, the position of the silicon probes was gradually adjusted along the dorsal-ventral axis until the pyramidal cell layer SWR events were recorded by uppermost region of the silicon probe. Once positioned, the probes remained stable across various recording sessions. For tetrode recordings, the tetrodes were individually moved from their original post-surgery location to the CA1 pyramidal layer, which could be distinctly identified by the pronounced presence of SWR events. Before beginning the recordings each day, the position of the tetrodes was fine-tuned to optimize both the clarity and the number of spike waveforms, based on visual assessment. Moreover, tetrodes targeting layers ventral from the CA1 pyramidal layer, or the dentate gyrus were moved downwards until they reached their intended target (see Tetrode feature-based placement section for details). Once the tetrodes were positioned for that recording day, we allowed for a 90-minute break before commencing the first recording session, ensuring sufficient time for the tissue to adjust. As a day of recording concluded, the tetrodes located in the pyramidal layer were cautiously retracted by about 125μm in the direction of the stratum oriens.

#### Laser stimulation

Laser stimulation was performed during the final two sessions (a 15-minute exploration and a 20–30-minute sleep/rest) ending the recording day. Light (wavelength, 473 nm; intensity, 400–600 μW; Crystal Laser model CL473-100, Laser 2000 Ltd., Ringstead, UK) was delivered through the 200 μm diameter optic fibers (Doric Lenses Inc., Quebec, Canada) combined with the 14 independently moveable tetrodes, as described previously. The stimulation protocol consisted of 1-ms pulses administered at intervals ranging from 1 to 4 seconds. For the analysis in Figure 6E, data from these sleep and awake sessions were combined.

#### Multichannel data acquisition, position tracking, and laser pulses

The extracellular signals from each recording channel were amplified, multiplexed, and digitized using a single integrated circuit located on the head of the animal (RHD2164, Intan Technologies; http://intantech.com/products_RHD2000.html; pass band 0.09 Hz to 7.60 kHz). The amplified and filtered electrophysiological signals were digitized at 20 kHz and saved to disk along with the synchronization signals (transistor-transistor logic digital pulses) reporting the animal’s position tracking and light pulses. The location of the animal was tracked using three differently colored LED clusters attached to the electrode casing and captured at 39 frames per second by an overhead color camera (https://github.com/kevin-allen/positrack/wiki).

#### Local field potential signals

LFP signals were processed by first applying an anti-aliasing filter (8^th^-order Chebyshev type I filter) to the wide band signals sampled at 20kHz. These signals were then downsampled to 1,250Hz using the decimate function from the signal submodule of Scipy.

#### Detection of SWR events

For SWR event detection, the LFPs were initially referenced against a channel where CA1 ripples were not observed. The resulting differential signal was filtered for the ripple band (80–250 Hz, 4th-order Butterworth filter) and for a control high-frequency band (200–500 Hz, 4th-order Butterworth filter). Instantaneous envelopes and phases for all ripple analyses were computed using the Hilbert transform. For tetrode recordings where all tetrodes exhibited ripples, no referencing was applied.

Candidate SWR events were identified when the peaks of the ripple band envelope exceeded a threshold set at five times its overall median value. If multiple peaks occurred within a 20-ms timeframe, only the highest peak was retained. The onset and offset of each candidate event were defined as the points where the envelope dropped below half the detection threshold. The number of ripple cycles within each event was calculated as the difference between the unwrapped phase at the offset and the onset. For example, an unwrapped phase difference of 1800° from onset to offset corresponds to five cycles, as 1800/360=5. The event’s mean frequency was calculated by dividing the number of cycles by its duration in seconds. For example, an event with 6.75 cycles lasting 50 ms would have a mean frequency of 135 Hz (6.75/0.05). Finally, each candidate event underwent a series of checks: (1) The ripple band power (derived from squaring the mean ripple amplitude) in the detection channel should exceed twice the magnitude obtained for the reference channel. This confirms that the detected events in the differential signal had a stronger presence in the detection channel; (2) The mean frequency of the event should be above 100 Hz; (3) The event had to include at least four complete ripple cycles; (4) The ripple band power had to be at least twice that of the control high-frequency band.

#### Determination of the reference CA1 pyramidal layer channel

For recordings, a single reference channel was used to analyze SWR events and theta oscillations. The reference channel was defined as the one with the highest ripple band score, calculated as the power in the ripple band (100–250 Hz) divided by the power in a surrounding frequency band (70–300 Hz). Power in each band was estimated using a Welch spectrum with 4-second Hann windows and 50% overlap.

#### Extraction of theta oscillations from LFPs

Theta oscillations were extracted from LFPs using the masked Empirical Mode Decomposition method ^120,124,125^. The mask sift procedure was employed with mask frequencies set to 350, 200, 70, 40, 30, and 7 Hz, following parameters optimized by Quinn et al.^92^ based on Fosso and Molinas^126^. The amplitude of each mask was set to three times the standard deviation of the input signal. This procedure decomposes each LFP signal into oscillatory components, termed intrinsic mode functions (IMFs), ordered from higher to lower frequency components. Using the parameters above, the process yielded six IMFs and a residue, with IMF-6 effectively isolating theta oscillations.

To delineate individual theta cycles, we first identified the peaks (local maxima) and troughs (local minima) of the theta IMF as derived previously. The residue of the LFP, not captured by the first six IMFs, was defined as the lower-frequency component of the signal, and its envelope was used as an amplitude threshold for retaining peaks and troughs in the subsequent step. Each peak-trough-peak sequence was then defined as a candidate theta cycle. Valid cycles were those with peak-trough and trough-peak intervals falling within 31 to 100 ms (corresponding to half the period of cycles with frequencies ranging from ~16 to 4 Hz). Additionally, peak-to-peak intervals had to range between 71 ms (~14 Hz) and 200 ms (~5 Hz). For each validated cycle, six control points were identified: the zero-crossing preceding the first peak, the peak itself, the zero-crossing following the peak, the trough, and the zero-crossing after the trough. The instantaneous theta phase for each timestamp was then computed using linear interpolation between these control points ^46,65^.

#### Current source density analysis

In this study, we applied CSD analysis ^61,127^ to the event-triggered averages from LFP recordings captured via linear silicon probes. These averages were calculated by aligning LFP signal intervals to event timestamps. For example, when analyzing theta oscillations, averages were centered around the descending zero-crossings of detected theta cycles. Once these averages were established for specific events, the current source density signal at channel *n* and a given time point was computed as:

$$CSD_{n}=-\left(LFP_{n-1}-2\cdot LFP_{n}+LFP_{n+1}\right)$$

where, *n−1* and *n+1* refer to the channels immediately above and below *n*. To standardize the spatial resolution of CSDs across silicon probes with varying channel spacings, Gaussian kernel smoothing was applied with a standard deviation of 50 μm.

#### Dentate spikes detection and classification

Dentate spikes in silicon probe recordings were identified during sleep/rest periods, as they predominantly occur in these brain states ^12,128^. LFPs from the dentate gyrus region were initially referenced by subtracting signals from a channel located at the top of the probe, typically positioned in the cortex or the upper oriens/alveus region. The resulting differential signal was then filtered within a frequency range of 1–200 Hz using a 4th-order Butterworth filter. Peaks in the filtered differential signal exceeding a threshold of seven times the median absolute value were identified as candidate dentate spike events. To eliminate high-amplitude artifacts, candidates with peaks exceeding a threshold—defined as the 75th percentile of the peak distribution plus 20 times its interquartile range—were excluded. This detection procedure was applied to channels within the dentate gyrus region, identified through visual inspection, and events from these channels were subsequently combined. If multiple events were detected within a 50-ms window, only the event with the highest peak was retained.

We applied PCA to dentate spikes to classify them into DS1 and DS2, based on established findings that these subtypes reflect distinct laminar patterns in the hippocampus^12,59^. More specifically, we analyzed each event by computing the CSD from the pyramidal layer channel to the deepest dentate gyrus channel at its peak. Principal Component Analysis (PCA) was then applied to these CSD vectors, projecting each individual CSD onto the first principal component. The first principal component of the CSD across all detected DS events effectively distinguished these subtypes by identifying the location of their sources and sinks. Classification was performed by applying a 2-component Gaussian Mixture Model (GMM) to the first principal component projections. In recordings that included both the molecular and granular layers of the dentate gyrus, this method consistently identified two distinct CSD patterns, each with primary sinks at different positions within the dentate gyrus channels. The pattern with its primary sink positioned further from the hilus was defined as DS1, while DS2 was assigned to the pattern with its sink closer to the hilus. If the GMM-defined classes revealed identical primary sinks, all dentate spike events were labeled as DS1, indicating the absence of a granular layer channel in the recordings.

#### Alignment for obtaining mean waveforms from LFPs

Throughout this manuscript, when computing triggered averages and mean waveforms, we aligned SWR events to the peak of the ripple envelope and DS events to the peak of the DS signal in the LFP. In contrast, theta oscillations are composed of continuous cycles, making the choice of a reference point less obvious. Although peaks or troughs may seem like natural candidates, aligning to them can introduce distortions. Aligning to peaks sharpens the average at that point but increases jitter at the trough due to variability in cycle duration. To minimize such distortions, we used the descending zero crossing of the theta oscillations recorded in the pyramidal layer as the reference point.

#### LFP feature embedding

The silicon probe recording dataset was used to construct the LFP feature space (“Hipp-LFP-embedding”; see Key Resources Table). For each mouse, sharp-wave and theta waveforms were computed for each recording channel, spanning from the CA1 pyramidal layer to the DG granular layer (Figure S2A). These waveforms represent the average raw LFP signals for individual channels, centered around either the ripple power peaks or the descending zero-crossings of theta (see section above). Theta and SWR waveforms were extracted using different window sizes to account for their distinct durations. Given that theta oscillations have a dominant frequency of ~8 Hz, we used a 150-ms window to capture their full cycle waveform with a margin. In contrast, SWR-related waveforms were extracted using a 500-ms window to encompass their full average deflection around ripple envelope peaks. To ensure uniformity across mice, channels were sampled at 50 μm steps (40 μm for mice with 20 μm-spaced channels; see Table S1). Each waveform was individually z-scored to emphasize waveform shapes rather than relative amplitudes across channels. Concatenating SWR and theta waveforms directly would lead to an imbalance in feature dimensionality, as SWR windows contain over three times more LFP data points than theta windows. To address this, we applied PCA separately to each waveform type and retained the first four principal components from each, ensuring balanced dimensionality while preserving most of the variance for the embedding input (Figure S2B).

Using these principal component projections, the embedding was computed with the Isomap method from the manifold submodule of scikit-learn, using parameters n_neighbors=15 and n_components=2. We used Isomap because our goal was to obtain a low-dimensional representation in which Euclidean distances reflect geodesic distances in the original space. Given our goal was to extract a trajectory mapping the evolution of theta and SWR waveforms across anatomical position, preserving global structure was essential. Other methods, such as t-SNE and UMAP, prioritize local structure and may distort long-range relationships. Although UMAP parameters can be tuned to better capture global features (for example, by increasing the number of neighbors) its primary goal is the preservation of local structure. Isomap, by contrast, is explicitly designed to preserve global geometry, making it a more natural choice for capturing the continuous, layer-dependent structure in our LFP data.

For constructing the k-nearest neighbor graph in Isomap, we used n_neighbors=15, ensuring a balance between preserving local structure and maintaining global relationships. Given our dataset size (136 channels in total, ~22.67 channels per mouse), this choice ensures that each point connects to a meaningful proportion of the dataset. Empirical testing confirmed that performance remained stable within a range of n_neighbors = 10–20, supporting the robustness of this choice. For computing geodesic distances in Isomap, we used Dijkstra’s algorithm, as it ensures computational efficiency and is the standard choice for sparse graphs. We used a 2D embedding as it captures 91.5% of the variance in the transformed data (SWR and theta waveforms after PCA). This choice also enhances interpretability, as 2D embeddings allow clear visualization. To further validate this, we evaluated classification performance in terms of mutual information (as in Figure 1H) while varying the number of Isomap dimensions. We found that performance peaked at 2–3 dimensions, confirming that additional dimensions do not provide non-redundant information for layer discrimination.

We next derived a trajectory connecting layer centers. To enhance resolution between distantly spaced clusters in the feature space (e.g., radiatum and lacunosum-moleculare), intermediate points were added before interpolation. The number of intermediate points between successive layer pairs was calculated by dividing the distance between points in the 2-D feature space by 20. Using this count, we selected an equivalent number of equidistant channels in the anatomical space for each mouse. Subsequently, averages for each control point (layer coordinates and intermediate points) were calculated across all mice. The final trajectory (Figure 1F, black trace) was determined by applying quadratic interpolation to these control points.

#### Trajectory consistency across recording days and mice

We computed Fréchet distances to quantify trajectory similarity within and across animals. The similarity between trajectories was defined as 10 divided by their Fréchet distance (the scaling factor of 10 was merely used to bring values closer to 1 for visualization). To assess the significance of the observed similarities, we compared them to a null distribution generated from surrogate trajectories (Figure 1G). Specifically, we (1) computed the Fourier transform of each trajectory, (2) applied circular shifts to the phases of its frequency components, and (3) reconstructed the trajectory using the inverse Fourier transform. Unlike simpler methods that randomly reorder trajectory channels, this Fourier-based surrogate procedure preserves spectral content and mean position, generating more realistic trajectories while selectively disrupting their precise structure. We computed 10,000 surrogate similarities for each trajectory pair. To compile group results, we normalized the observed similarity values relative to their corresponding surrogate distributions as:

$$s_{z}=\frac{s-\mu_{surrogate\;}}{\sigma_{surrogate\;}}$$

where *s* is the observed similarity of a trajectory pair, *μ*_*surrogate*_ is the mean of the surrogate distribution (i.e., the expected similarity for surrogate trajectories with matched spectral characteristics), and *σ*_*surrogate*_ is the standard deviation of the surrogate distribution.

#### Classification of layers from feature space projections

Layer separability within the feature embedding was assessed using cross-validated classification. A leave-one-out methodology was employed, where each classifier was trained on all data points except one, which was reserved for testing. Thus, each mouse contributed a single layer channel, meaning the classifiers primarily relied on datasets from other mice for layer decoding. A k-neighbor classifier was used (implemented using the ‘neighbors’ submodule of sklearn; parameters: weights=‘uniform’, algorithm=‘auto’, and k=4). The choice of k=4 was determined by the smallest sample size across layers.

#### Embedding projection variability as function of sample size

To evaluate how the number of network events used to compute average LFP waveforms affects the embedding, we performed a subsampling analysis for both SWR events and theta cycles. LFP waveforms were obtained as before, but using randomly selected subsets of events (either theta cycles or SWR events). Importantly, subsets were always composed of consecutive events rather than uniformly sampled across the session, allowing us to have an idea of how much continuous data is required for projections to converge. For each event count, we generated up to 10,000 projected data points (e.g., if 5,000 events were available, one projection was generated for each possible consecutive subset). Each average data point was projected onto the same embedding constructed previously (Figure 1). When subsampling SWR events, all available theta cycles were used (and vice versa). Figures 7A and 7C show the resulting projection histograms across different sample sizes for representative layers and recording sessions. To quantify projection variability (spread) for each layer and subsample size, we computed the square root of the determinant of the 2D covariance matrix of the projected points (Figure 7B,D, top panels). To assess how subsampling affected classification performance, we applied the same k-nearest neighbors classifier trained in Figure 1H to assign a layer label to each projection. The probability of correct classification is shown in the bottom panels of Figures 7B and 7D for each layer as a function of the number of SWR events or theta cycles, respectively. For this classification analysis, we grouped l-m, hf, and om layers into a single category, as they were not distinguishable in the original classifier results (Figure 1H).

#### Supra-theta wavelet spectrograms

Spectrograms were generated using the complex Morlet Wavelet Transform. A range of 24 logarithmically spaced frequencies, spanning from 18 Hz to 310 Hz, was used unless stated otherwise. Each wavelet kernel was L1-normalized, ensuring that the sum of the absolute values of its elements equals 1. This normalization standardizes the wavelet kernel's magnitude, ensuring that differences in amplitude across frequency components reflect the input signal rather than the kernel's scale.

#### Gamma filtering and instantaneous phase and amplitude

For amplitude and phase analyses of specific gamma oscillations, LFP signals were filtered within the relevant bands using a 4th-order Butterworth filter. The cutoff frequencies were defined as follows: 20–45 Hz for slow gamma, 50–100 Hz for mid gamma, and 100–250 Hz for fast gamma. Instantaneous amplitude and phase were then computed using the Hilbert transform.

#### ICA-based extraction of fast gamma oscillations

Isolating fast gamma oscillations poses a challenge due to their relatively low amplitude, especially in channels that exhibit higher amplitude mid gamma oscillations. Any overlap with mid gamma oscillations can contaminate the signal filtered for the fast gamma range, given the comparable magnitude of the high-frequency tail of the mid gamma spectrum and the fast gamma oscillation itself. To address this, we adopted an ICA-based method inspired in previous work ^129^. This approach leverages spatial data from silicon probe recordings to differentiate fast gamma from mid gamma oscillations. Specifically, ICA was applied to LFP traces extending 200 μm around a targeted channel with fast gamma interest (e.g., pyramidal, distal radiatum, mid moleculare, or granular layer), and we extracted independent components with peak frequencies above 100Hz. We used the amplitude of such signals for the fast gammas in Figure 2B.

#### Determination of gamma main frequencies

To identify the primary frequency components of each gamma oscillation, we first detected the strongest bursts within theta cycles by locating envelope peaks in the appropriate gamma band during individual cycles and retaining only the top quartile of these peaks. For fast gammas, see ICA-based extraction of fast gamma oscillations section above. Using these peaks, mean spectra were generated by averaging wavelet spectrograms within a 40-ms window centered on the burst peaks. Before averaging across mice (Figure 2C), the triggered average spectrogram for each mouse was normalized by its overall standard deviation.

#### Gamma speed modulation

To assess whether the amplitude of different gamma oscillations across layers was modulated by speed, we quantified speed modulation separately for each of the six identified gamma bands. For each recording day, we divided theta cycles into six equally populated speed quantiles during open field exploration. For each quantile, we computed the mean amplitude of each gamma rhythm, yielding a six-bin amplitude-versus-speed curve per gamma and recording day. To normalize across sessions, amplitude values were divided by their mean across bins and log2-transformed. Speed modulation was then quantified as the slope of a linear regression fit to the normalized amplitude values across the six speed quantiles.

#### Tetrode feature-based placement

To target layers below CA1 pyramidal cells with tetrodes, we employed a stepwise, gradual lowering approach. Short sessions (typically 5–10 minutes) were recorded during both sleep and wakefulness to project LFP features onto the embedding (as depicted in Figure 1D,E, and Figure S2). Tetrodes were adjusted by no more than ~60 μm within a 20-minute interval. Before recording sessions for spiking activity analysis, we waited 90 minutes after the last tetrode adjustment to allow tissue accommodation and optimize signal stability.

For the analysis of spike correlations with specific gamma oscillations in the tetrode data, we focused on the appropriate layer corresponding to each rhythm (Figure 4, 5 and 6). Slow gamma was consistently analyzed from radiatum channels, mid gamma from lacunosum-moleculare channels, and each fast gamma from its respective layer.

#### Spike detection and unit isolation

Spike sorting and unit isolation were performed using an automated clustering pipeline implemented via Kilosort within the SpikeForest framework ^122,123^. For tetrode data, Kilosort restricted templates to channels within each tetrode bundle while masking all other recording channels. The resulting clusters were manually verified by inspecting cross-channel spike waveforms, auto-correlation histograms, and cross-correlation histograms. Units included in the analyses exhibited stable spike waveforms throughout the entire recording day.

#### Principal cell versus interneuron classification

To assess waveform consistency for each unit, we analyzed the waveform with the maximum amplitude across tetrode channels within each cluster. The primary objective was to evaluate the magnitude of a unit’s mean waveform amplitude relative to the standard deviation of all its spikes. This metric, referred to as the waveform score, was defined as:

$$wv_{score\;}=\sqrt{\sum_{i=1}^{n}\frac{{\left(w_{i}/\sigma_{wi}\right)}^{2}}{n}}$$

where *w*_*i*_ is the value of the mean waveform at sample *i*, *σ_wi_* is the standard deviation at sample *i* across all spikes, and n is the number of waveform samples (in this context, n=32). This metric quantifies the relative magnitude of the mean waveform amplitude compared to spike-to-spike variability. Clusters with a waveform score below 0.75 or a refractory period violation exceeding 2% (measured as the proportion of intervals shorter than 2 ms in the ISI distribution) were classified as multi-units and excluded from further analyses. Additionally, clusters with positive spikes were disregarded, as these likely originated from non-somatic spikes ^130^. Only units meeting all these criteria were considered well-isolated and included in subsequent analyses.

Finally, units were categorized as putative interneurons or pyramidal cells based on the width of their waveform, measured by trough-to-peak latency. To improve resolution, the 32 waveform points sampled at 20 kHz were upsampled by a factor of 100 using quadratic interpolation (via the interpolate function from scipy). In a previous dataset of ~4,000 well-isolated neurons, a bimodal distribution of trough-to-peak latency was observed. This distribution was modeled using a 1-dimensional, 2-component Gaussian Mixture Model (GMM) from scikit-learn, and the classification threshold was set at the intersection of the two Gaussian components. Applying this threshold to the current dataset, units with latencies above the threshold were classified as putative pyramidal cells, and those below as putative interneurons.

#### Gamma and ripple trough triggered averages

In Figures 4B, 4E, 4H, 5E, and 6H, mean spiking activity and LFP signals were computed around the most prominent troughs of the targeted gamma rhythms or ripples. LFP signals were first filtered within the relevant frequency range for the specific layer, as described earlier. For gamma oscillations, the deepest trough within each theta cycle was identified, and only the top quartile of these troughs (in terms of amplitude depth) was used for triggered-average analyses. By aligning spike trains or LFP signals to a single gamma trough per theta cycle, this analysis minimizes the risk of artificially inflating gamma rhythmicity by mitigating biases in the triggered averages that could result from the band-pass filtering process. Similarly, for ripple-triggered averages, pyramidal LFPs were filtered within the 100–250 Hz range, and the deepest ripple troughs from each SWR event were used for alignment.

#### Spike to phase coherence analysis

Theta oscillations are asymmetrical (non-linear), leading to an uneven distribution of phases. In recordings from the pyramidal layer, the rising phase is shorter than the falling phase, resulting in an overrepresentation of the latter in the theta phase distribution. This asymmetry can bias coherence analyses by artificially increasing the association of spikes with the longer falling phase. To correct for this, we normalized the spike-phase coherence by accounting for the likelihood of a spike occurring in each theta phase bin. This was achieved by dividing the distribution of theta phases associated with neuron spikes by the overall theta phase distribution. Both distributions were represented as histograms with 64 equally spaced phase bins. Spike-to-phase coherence was then quantified using the mean vector length, where each phase bin center was weighted by its corresponding probability.

For gamma phase coherence, the methodology mirrored that of the theta analysis but included a modification to account for the transient nature of gamma oscillations. A threshold based on the gamma envelope was introduced, selecting only spikes and gamma phases where the envelope exceeded its 75th percentile. This approach ensured the data reflected periods with genuine gamma activity in the signal. For ripple phase coherence, only phases between the onset and offset of the events were considered.

For a given oscillation, phase coherence analysis was restricted to units with at least 250 spikes within that oscillation periods. To evaluate significance, a spike shift control was employed. For each neuron, spikes were shifted to random time points matching the original theta phase and gamma amplitude of their respective time points ^46^. This surrogate process was repeated to generate a null hypothesis distribution comprising 10,000 surrogate values. The p-value for each neuron was calculated as the proportion of surrogate values that matched or exceeded the observed coherence value, representing the likelihood of that level of coupling occurring by chance. Only units with significant coupling (p < 0.01) were included for mean phase estimation.

Of note, when estimating the mean firing phases of neurons, all analyses were performed in the continuous phase domain as described above. Alignments to specific reference points (e.g., the descending zero-crossing of theta oscillations) were used only for triggered averages of instantaneous firing rates and were not used for quantifying mean phases or spike-to-phase coherence.

#### Classification of deep and superficial principal cells

In the analyses shown in Figure 5, principal cells were categorized as deep or superficial based on the LFP features recorded by the tetrodes. The classification was determined using the linearized projection of these features onto the feature embedding (Figure 1E, right). The distribution of these projections across the dataset was distinctly bimodal. To model this, we applied a 1-dimensional, 2-component Gaussian Mixture Model (GMM) using scikit-learn. The resulting Gaussian distributions, superimposed on the projection data, are shown in Figure 5B. The classification threshold for deep versus superficial cells was set at the intersection of these Gaussians, approximately corresponding to a value of 5.5.

#### Anatomy

After completion of the electrophysiological recordings, mice were anaesthetized with pentobarbital and transcardially perfused with 4% PFA (150ml, 8 – 10 ml/min). Subsequently, the heads with implanted tetrode microdrives were post-fixed overnight at 4°C. The next day, the implanted microdrives were removed and the brains were resected followed by post-fixation in 4% PFA for 2 hours. Then brains were either sectioned at 50 micrometers on a vibratome (Leica Microsystems VT1000S) or embedded in gelatine and cryoprotected to be sectioned on a freezing microtome (Epredia HM 450 with a Physitemp BFS-40MPA freezing stage). For embedding, brains were first incubated overnight in 10% sucrose 0.1M Phosphate Buffer solution at 4°C. Then embedded in gelatine (12% gelatine / 10% sucrose), stored in 30% sucrose 0.1M PB overnight at 4°C for cryoprotection, and sectioned the following day utilizing the freezing sliding microtome ^131^. Sections were stained with 4',6-diamidino-2-phenylindole (DAPI; 0.5 μg/ml, Sigma-Aldrich, cat# D8417) diluted in PB to label cell nuclei, mounted, and cover-slipped with Vectashield mounting medium. Images were acquired using a Zeiss LSM 880 confocal microscope equipped with Plan-Apochromat 10x/0.45, 20x/0.8 objectives. DAPI and an empty channel were imaged using excitation wavelengths of 405, and either 458 or 633 nm.

## Quantification And Statistical Analysis

Data analyses were performed using Python 3.10 with the following packages: scikit-learn 1.2.2, NumPy 1.26.4, SciPy 1.14.1, Matplotlib 3.10.0, and Pandas 1.5.3.

Confidence intervals were calculated using a standard bootstrapping procedure. Original data points were resampled with replacement, and the mean was computed for each resample. This process was repeated 100,000 times to generate a bootstrap distribution of means. The 99% confidence interval was defined as the range between the 0.5th and 99.5th percentiles of this distribution. For bootstrap-based p-values, a null distribution of differences between resampled groups was generated. The one-tailed p-value was calculated as the proportion of the null distribution with values equal to or exceeding the absolute observed mean difference. For two-tailed tests, this p-value was multiplied by 2. Permutation tests were used to assess the significance of differences between cell groups by comparing the observed mean difference to a null distribution generated through random reassignment of group labels. For example, to evaluate the mean rate difference between deep and superficial cells, cell labels were randomly shuffled, and the mean difference for each permutation was recorded. This process was repeated 100,000 times to create the null distribution, which represents the distribution of mean differences expected under random group assignments. For one-tailed tests, the p-value was computed as the proportion of the null distribution with values equal to or exceeding the observed mean difference. For two-tailed tests, the resulting one-tailed p-value was multiplied by 2.

To assess the significance of light-responsive cells in Figure 6E, we compared each cell’s firing rate in a 10-ms window after laser onset to its baseline rate measured over the 1 s preceding the laser. The observed firing rate increase (post-laser onset minus baseline) was calculated for each cell. Statistical significance was assessed using a permutation test, where baseline and post-laser onset firing rates were randomly swapped with a 50% probability in each trial, and the difference was recalculated. This process was repeated 100,000 times to generate a null distribution of firing rate differences expected by chance. A cell was considered significantly responsive if its observed increase exceeded the 99th percentile of this null distribution. To exclude small effect sizes, the post-laser onset firing rate also had to exceed the baseline mean by at least two standard deviations. Results were consistent across different baseline window sizes, ranging from 10 ms to 1000 ms.

## Supplementary Material

Supplemental Figures and Tables

## Data Availability

The dataset reported in this study is being used in ongoing projects and can be accessed under a data transfer agreement. We welcome enquiries for sharing it; please contact david.dupret@bndu.ox.ac.uk. The hippocampal LFP embedding analysis code has been deposited on GitHub and archived on Zenodo (https://doi.org/10.5281/zenodo.15275527) and is publicly available. Any additional information required to reanalyze the data reported in this paper is available from the lead contact upon request.
