# Supplementary material for: Spatio-temporal Organization of Network Activity Patterns in the Hippocampus
Source: Cell Rep. Author manuscript; Available in PMC 2025 Jun 9. (PMC7617751; doi:10.1016/j.celrep.2025.115808)
Supplement: Supplemental Figures and Tables [file EMS205926-supplement-Supplemental_Figures_and_Tables.pdf]

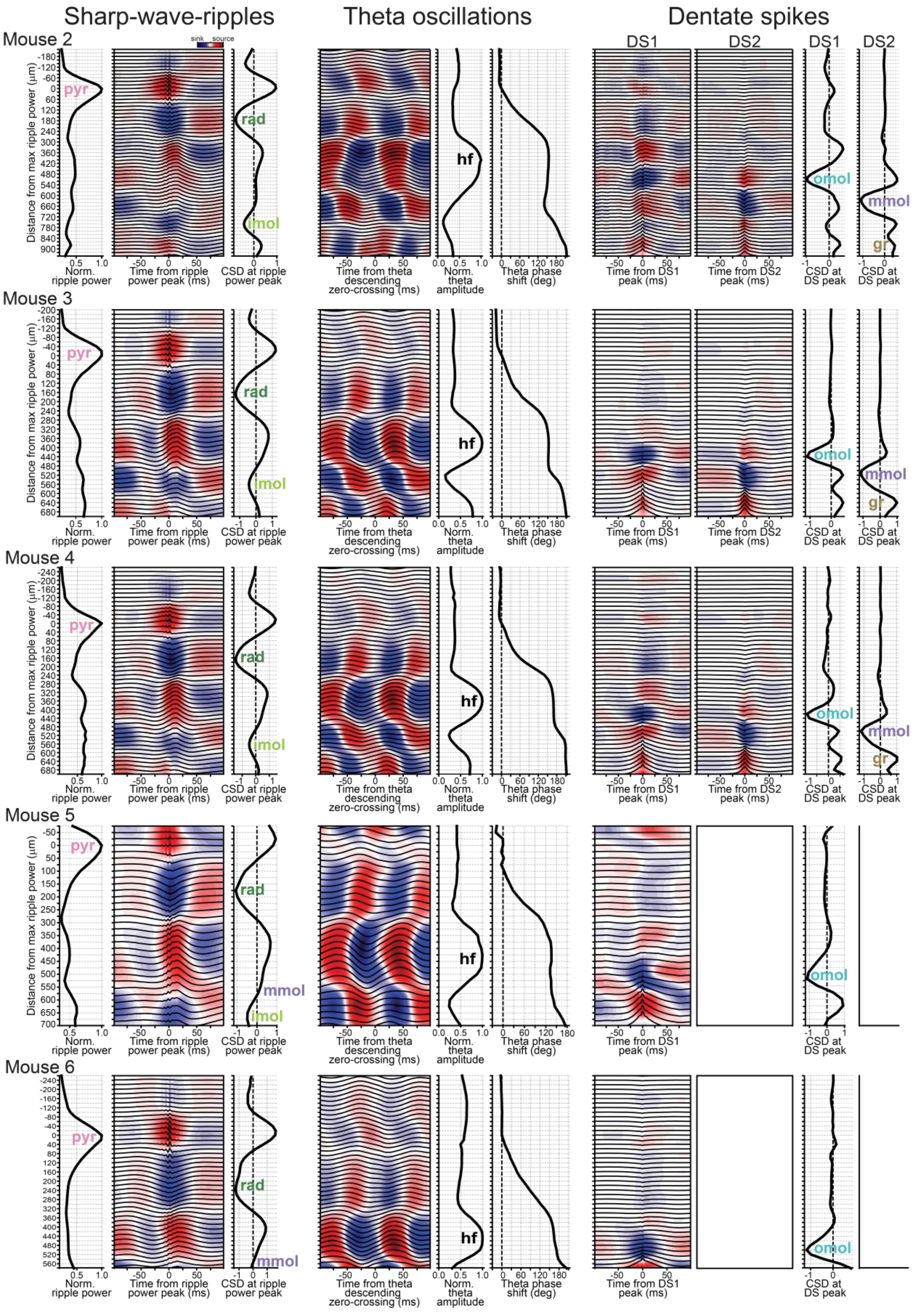

Sharp-wave-ripples
Theta oscillations
Dentate spikes
Mouse 2
Mouse 3
Mouse 4
Mouse 5
Mouse 6
sink
source
DS1
DS2
DS1
DS2
Distance from max ripple power (µm)
pyr
rad
lmol
mmol
hf
omol
gr
Norm. ripple power
Time from ripple power peak (ms)
CSD at ripple power peak
Time from theta descending zero-crossing (ms)
Norm. theta amplitude
Theta phase shift (deg)
Time from DS1 peak (ms)
Time from DS2 peak (ms)
CSD at DS peak

Figure S1. Identification of hippocampal layers using electrophysiological patterns in the silicon probe dataset. *Related to Figure 1.*

Activity profiles used for layer determination in mouse 2 to mouse 6 (see mouse 1 in Figure 1C). From left to right: The sharp-wave ripple panels display ripple power across layers, CSD analysis of LFPs aligned to ripple power peaks, and CSD values at the ripple power peak. The theta oscillation panels display the CSD for LFPs aligned to the pyramidal layer's descending zero-crossing, alongside normalized theta amplitude and phase shift across layers. The phase shifts are relative to the phase of the pyramidal layer. The dentate spike panels depict CSD analysis for DS1 and DS2 with their respective CSD values at their peak.

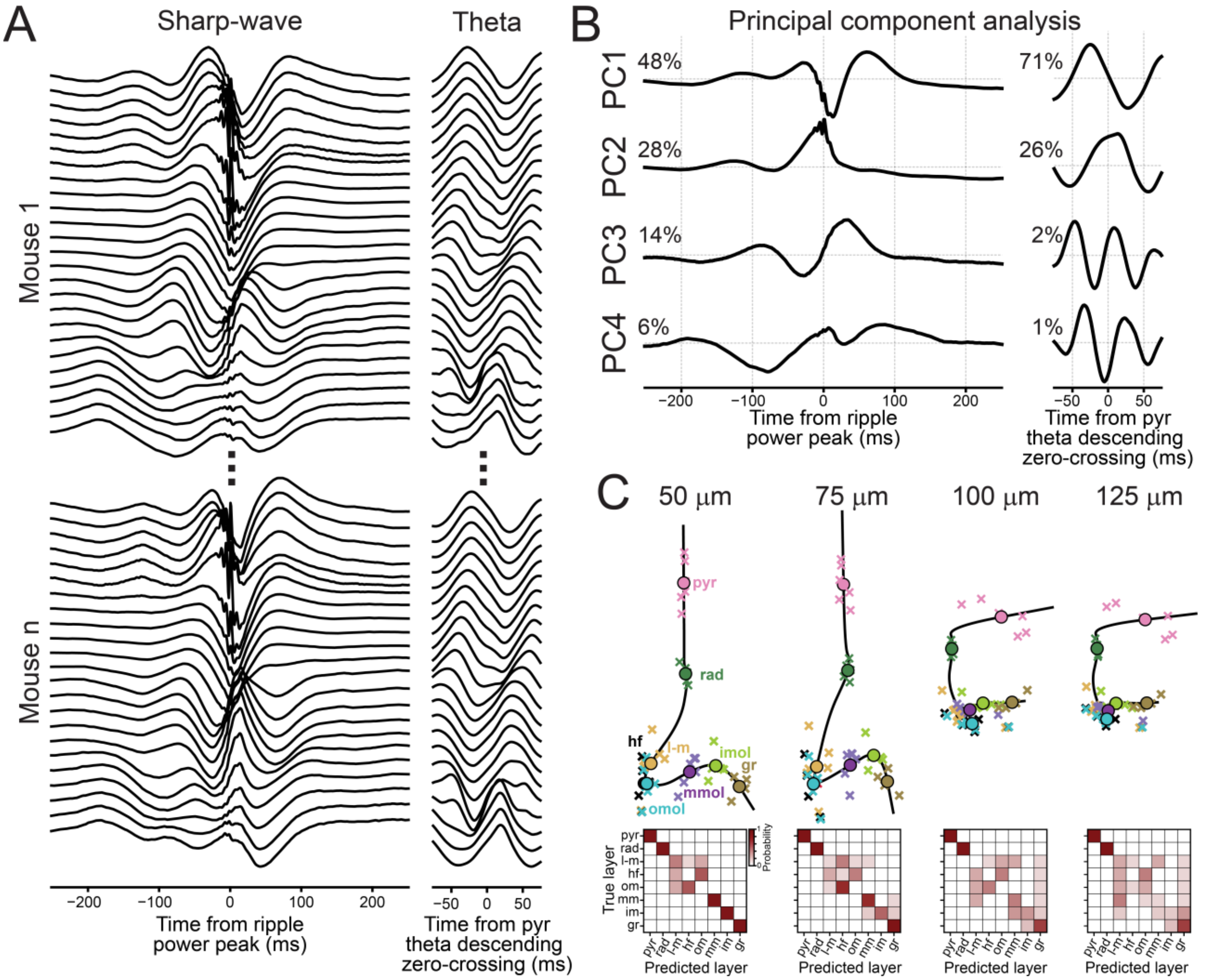

Figure S2. LFP-based feature embedding construction. ***Related to Figure 1.***

(**A**) LFP signals employed in defining the feature space. We extracted sharp-wave and theta waveforms from recording channels of the six mice with silicon probe implants. These channels span the dorsal hippocampus radial axis, from the CA1 pyramidal layer to the DG granular layer.

(**B**) We then applied principal component analysis to the sharp-wave and theta waveforms. Displayed are the first four principal components (PC 1 to PC 4) for each feature, along with their respective proportion of explained variance.

(**C**) Impact of anatomical sampling resolution on embedding construction. We recomputed the Isomap embedding as before, systematically increasing the spacing between used channels along the silicon probe shank. While the original embedding was computed using channels spaced at 50 µm intervals, we repeated the manifold construction and subsequent layer classification using coarser spacings of 75, 100, and 125 µm. Note that the channels used for CSD-based layer identification were preserved across all spacing conditions, as the CSD is only used to define ground-truth layer identities. Top panels: 2D embedding trajectories and layer projections for each spacing used. Bottom panels: Corresponding confusion matrices showing layer classification performance. Note that as sampling resolution decreases, the trajectory below stratum radiatum bends and collapses, approximating the l-m and dentate gyrus layer clusters. Further, at spacings above 100 µm, the trajectory bends bringing the CA1 pyramidal layer closer to the dentate gyrus granular layer. These distortions indicate that effective sampling along the laminar CA1-to-DG axis is necessary to construct a feature trajectory that accurately maps anatomical positions.

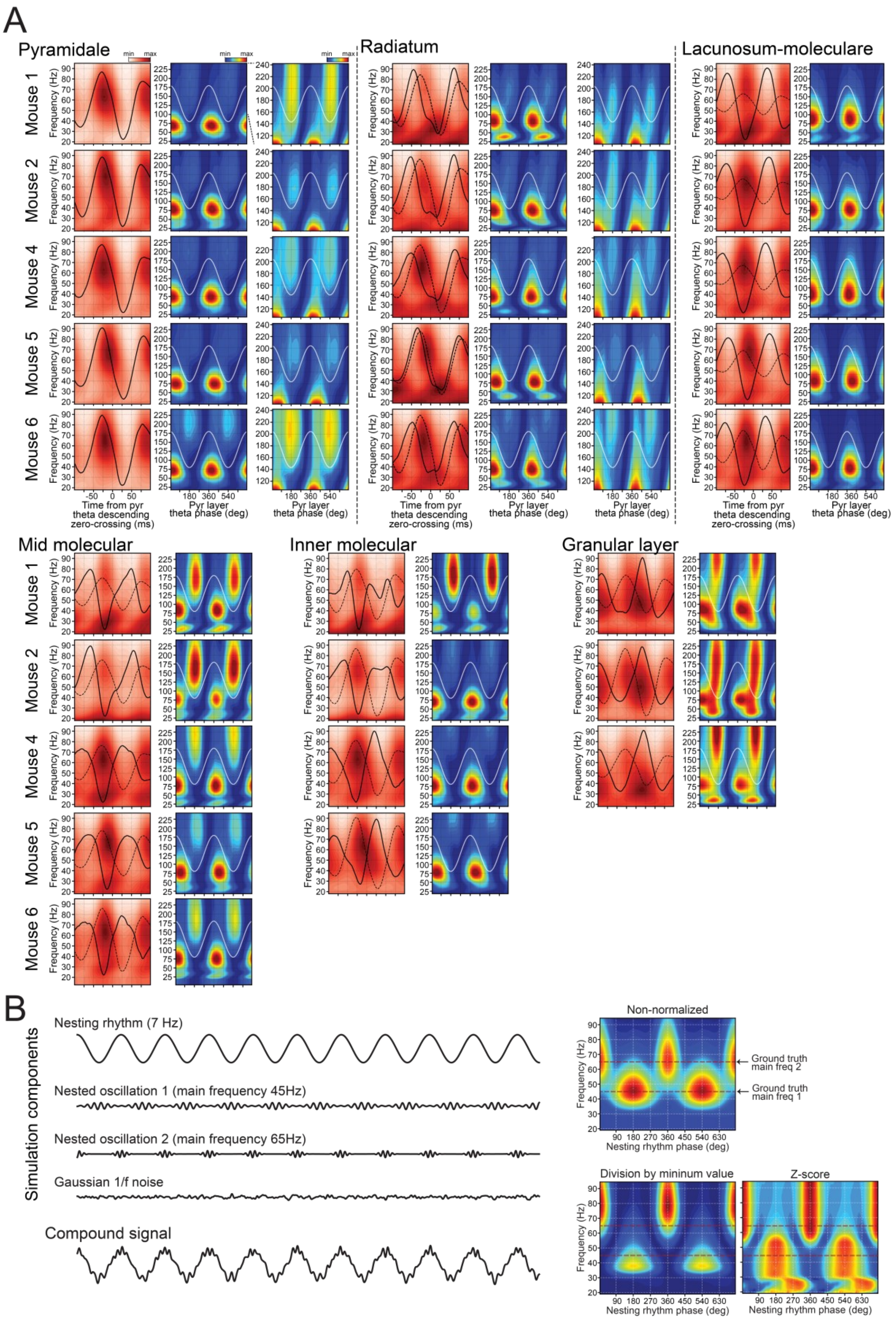

A
Pyramidale
Radiatum
Lacunosum-moleculare
Mouse 1
Mouse 2
Mouse 4
Mouse 5
Mouse 6
Frequency (Hz)
min max
Time from pyr theta descending zero-crossing (ms)
Pyr layer theta phase (deg)
Mid molecular
Inner molecular
Granular layer
B
Simulation components
Nesting rhythm (7 Hz)
Nested oscillation 1 (main frequency 45Hz)
Nested oscillation 2 (main frequency 65Hz)
Gaussian 1/f noise
Compound signal
Non-normalized
Ground truth main freq 2
Ground truth main freq 1
Nesting rhythm phase (deg)
Division by mininum value
Z-score

Figure S3. Cross-layer theta-nested gamma profiles for individual mice in the silicon probe dataset. *Related to Figure 2.*

(**A**) Shown are the theta nested-gamma profiles for the other mice in the silicon probe dataset (as in Figure 2). A zoomed-in view into the higher frequencies (> 100 Hz) is provided in the right-most panels for pyramidal and radiatum layers. This magnification distinctly reveals the presence of fast gamma oscillations, which can be partially masked by mid-gamma oscillations.

(**B**) Effect of spectrogram normalization on the frequency range for overlapping gamma components. Here, we used in silicon data simulation to assess the potential distortion in peak frequency caused by the normalizing frequencies independently. The left traces display the four simulation signal components: a nesting 7-Hz theta-like rhythm, two nested oscillations with known main frequencies, and Gaussian 1/f noise. The nested oscillations are emulated using cosines of defined ground truth frequencies (45 and 65 Hz), with an oscillatory envelope matching the nesting rhythm's frequency, producing phase-to-amplitude coupling. We then summed these components to generate a simulated compound signal. We finally constructed a theta-gamma-like profile of this compound signal (as in Figure 2). This was achieved without frequency amplitude normalization (top spectrogram), by normalizing each frequency based on its minimum value (bottom left spectrogram), and through z-scoring the amplitude of each frequency (bottom right spectrogram). Notably, the non-normalized version of these approaches offers a more precise portrayal of the foundational frequencies. Dividing frequencies by their minimal value tends to "push apart" the primary frequencies of slightly overlapping components. In contrast, z-scoring amplifies each component's spectrum tail considerably.

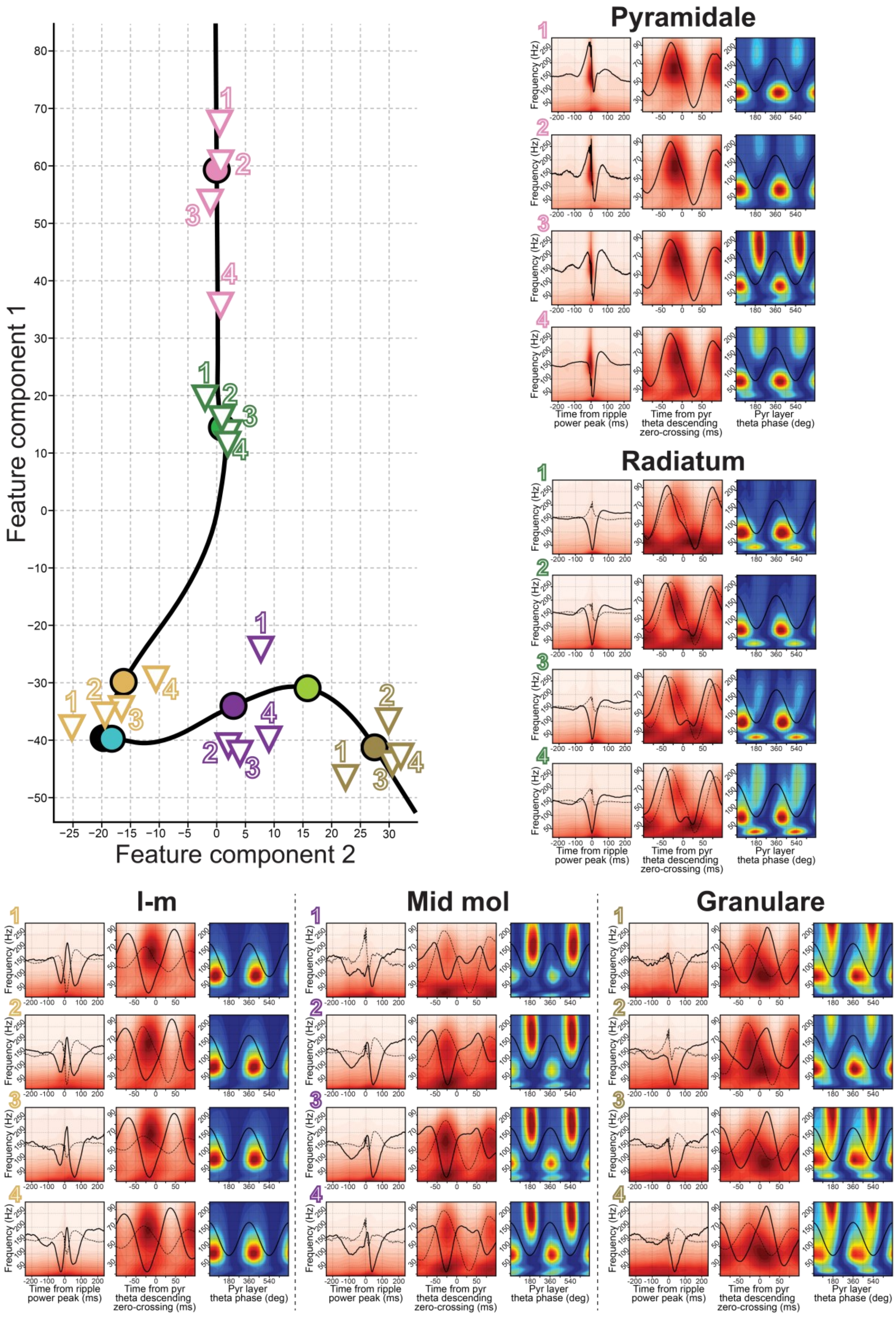

Pyramidale
Radiatum
l-m
Mid mol
Granulare
Feature component 1
Feature component 2
Frequency (Hz)
Time from ripple power peak (ms)
Time from pyr theta descending zero-crossing (ms)
Pyr layer theta phase (deg)

Figure S4. SWR and theta-gamma profiles from tetrode recordings along the CA1-DG axis. ***Related to Figure 3.***

This figure features four representative tetrodes, positioned across distinct layers of the CA1-DG axis, guided by the feature embedding. For each tetrode, shown are the SWR and theta-gamma profiles as in Figure 3.

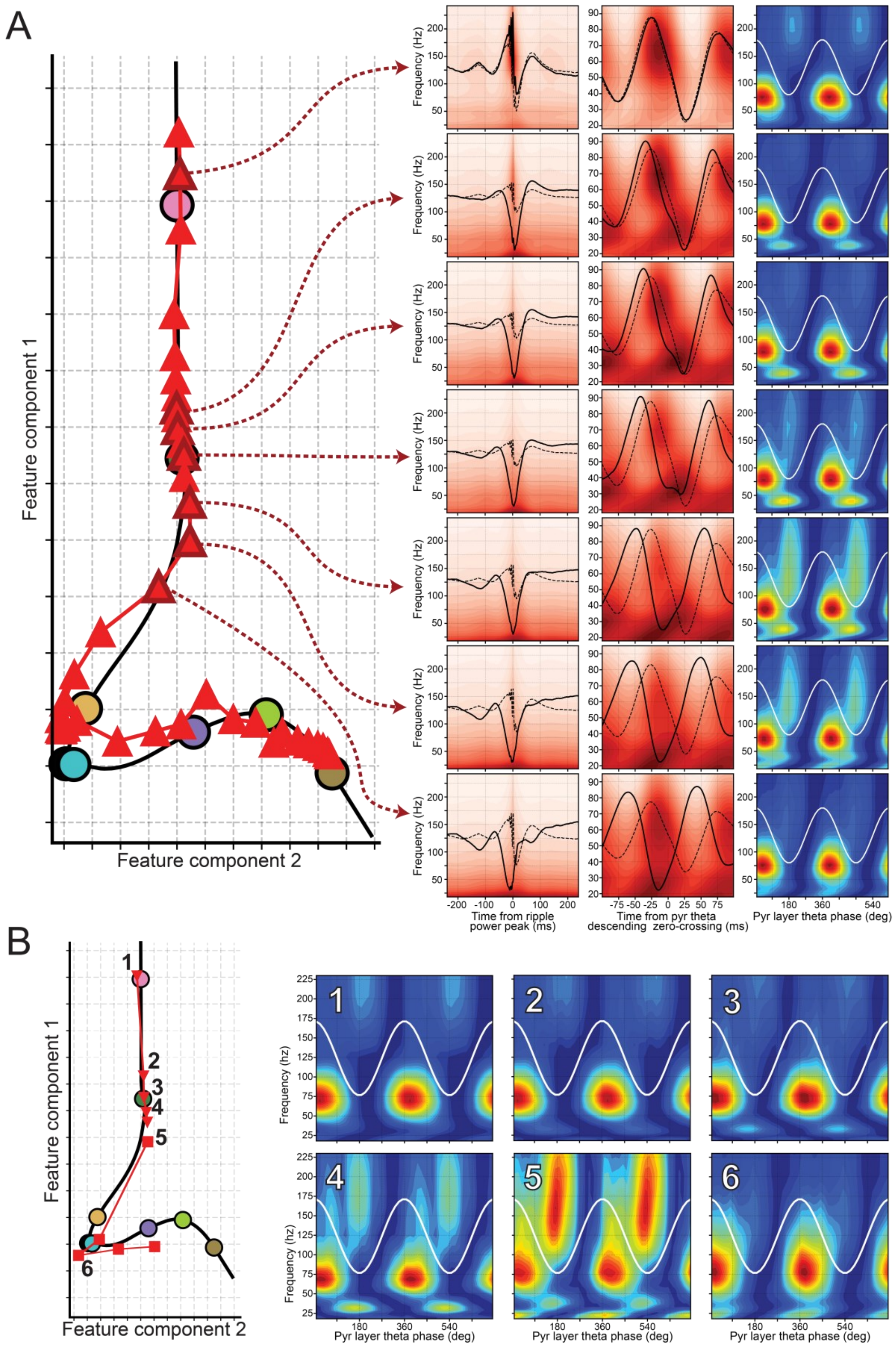
A
Feature component 1
Feature component 2
Frequency (Hz)
Time from ripple power peak (ms)
Time from pyr theta descending zero-crossing (ms)
Pyr layer theta phase (deg)
B
1
2
3
4
5
6
Frequency (hz)

Figure S5. Radiatum fast gamma is observed below the center of the radiatum layer. *Related to Figure 3.*

(**A**) The feature embedding projection of silicon probe channels (spaced at 20 mm) from a representative mouse is displayed. Accompanying this, the SWR and theta-gamma profiles of selected channels are presented on the right, with arrows indicating their respective positions. Notably, the radiatum fast gamma component (visible in the right-most panels showing the normalized theta-gamma profiles) is more pronounced in the recording channel located slightly below the center of the radiatum layer on the embedding.

(**B**) A depiction like A, but from a tetrode recording experiment as in Figure 3. The experiment began with a tetrode positioned in the pyramidal layer and then gradually moved towards the DG. Theta-gamma profiles for the sessions labeled on the left panel are detailed on the right. Remarkably, during session 5, with the tetrode positioned just below the radiatum center, the radiatum fast gamma component is at its peak clarity.

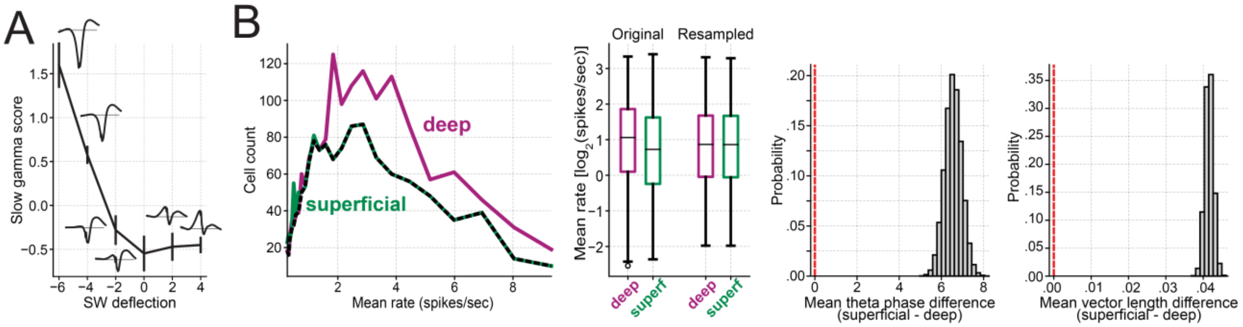

Figure S6. Gamma sharp-wave relationships and rate-matched principal cell analysis. *Related to Figure 4.*

(**A**) Shows the relation between slow gamma amplitude and sharp-wave depth. The slow gamma metric is measured as the amplitude between 25 and 45 Hz during the theta phase of 90 to 270 degrees, divided by its total amplitude above 18 Hz. Sharp-wave deflection is the value obtained from the mean sharp-wave waveform at the ripple power peak, divided by the waveform's standard deviation. Vertical bars on each data point denote the 99% confidence interval. Each data point is accompanied by insets that represent the average sharp-wave derived from the channels in their corresponding sharp-wave deflection bin.

(**B**) Shows the outline of rate-matched analysis for comparing between deep and superficial pyramidal sub-layer cells. The far-left panel shows histograms of average firing rates for each principal cell sub-population, computed over 25 log-spaced bins spanning from 0.25 to 10 spikes per second. The dashed line marks the lowest count across the distributions for each rate bin, guiding the histogram for resampled distributions. The next panel juxtaposes the original rate distributions with a representative resampled version. During resampling, cells are randomly chosen from each rate bin for a designated cell group, aligning with the counts highlighted by the dashed line in the far-left panel, thus assuring that resampled groups have equivalent mean rate distributions. The right-most panels show the distributions of differences in mean theta phase and mean vector length between deep and superficial principal cells across 100,000 resampling operations. These difference distributions consistently stand apart from zero demonstrating disparities in the mean theta phases and the strength of their couplings across the two cell sub-populations even when rate matched.

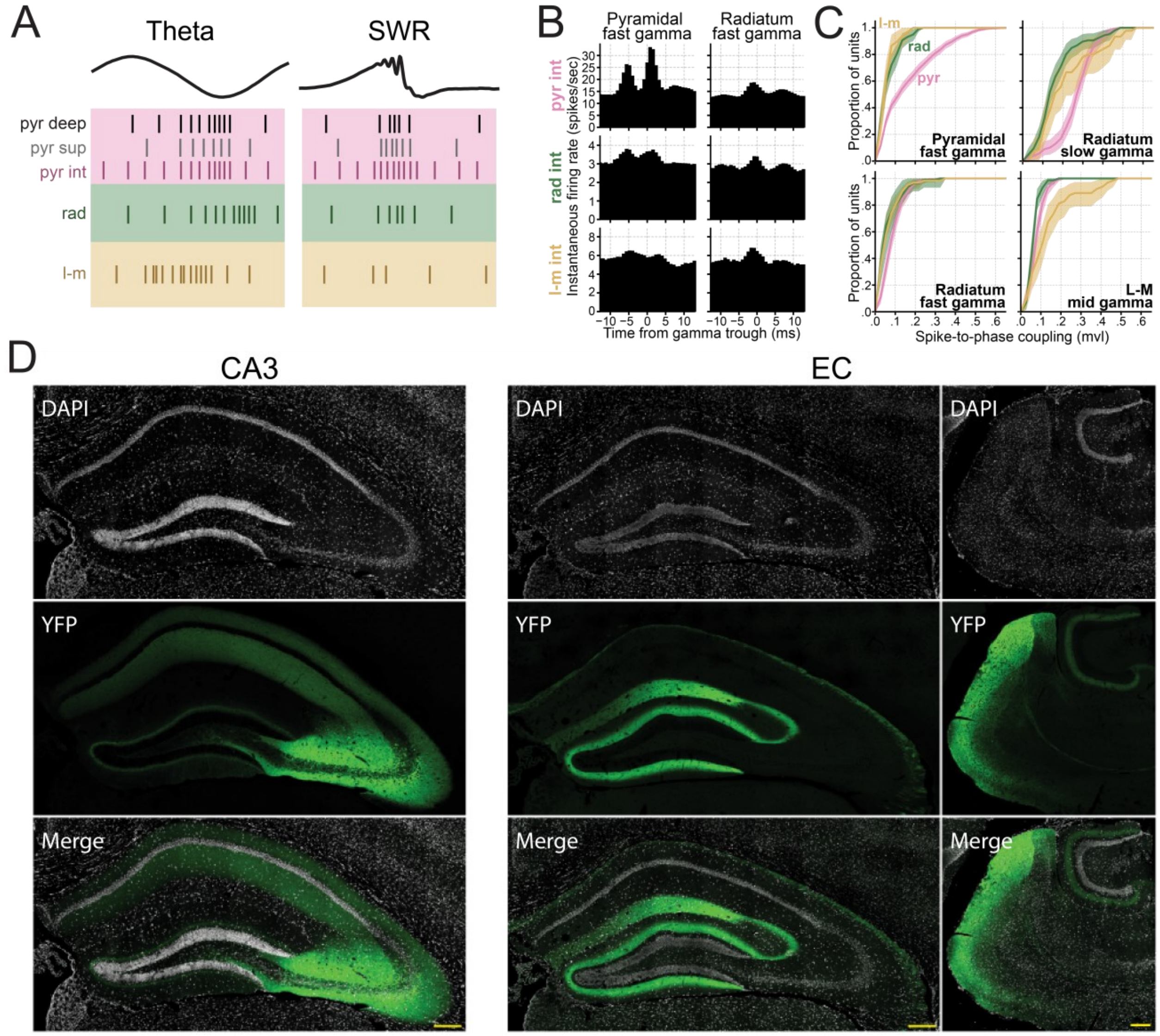

Figure S7. Layer-dependent firing behavior of CA1 interneuron. ***Related to Figure 6.***
(**A**) Summary schematic illustrating layer-specific firing patterns during theta oscillations (left) and SWRs (right) for different neuronal populations. Pyr deep, principal cells in deep CA1 pyramidal cell sublayer; Pyr sup, principal cells in superficial CA1 pyramidal cell sublayer; Pyr int, interneurons in CA1 pyramidal layer; rad, interneurons in CA1 radiatum layer; l-m, interneurons in CA1 lacunosum-moleculare layer.
(**B**) Same as Figure 6H but for pyramidal and radiatum fast gamma oscillations.
(**C**) Cumulative distribution of spike-to-phase coupling levels (mean vector length, mvl) for interneurons in the pyramidal, radiatum, and lacunosum-moleculare (l-m) layers for different gamma oscillations. For pyramidal fast gamma, mvl [mean (99% CI)] was: pyramidal 0.164 (0.152–0.177), radiatum 0.062 (0.05–0.074), and l-m 0.052 (0.041–0.065). Pyramidal interneurons exhibited significantly higher coupling than radiatum and l-m interneurons (both p <10-5), with no significant difference between radiatum and l-m interneurons ($p = 0.66$). For radiatum slow gamma, mvl was: pyramidal 0.264 (0.246–0.283), radiatum 0.161 (0.137–0.189), and l-m 0.212 (0.161–0.266). Pyramidal interneurons showed significantly higher coupling than radiatum ($p < 10^{-5}$) and l-m ($p < 0.01$) interneurons. For radiatum fast gamma, mvl was: pyramidal 0.085 (0.076–0.094), radiatum 0.061 (0.047–0.077), and l-m 0.060 (0.043–0.082). Pyramidal interneurons were significantly more coupled than radiatum ($p = 0.001$) and l-m ($p = 0.002$) interneurons. For l-m mid gamma, mvl was: pyramidal 0.075 (0.068–0.083), radiatum 0.060 (0.052–0.070), and l-m

0.132 (0.098–0.172). L-m interneurons exhibited significantly stronger coupling than both pyramidal and radiatum interneurons (both $p < 10^{-5}$). All reported p-values are from two-tailed pairwise permutation tests.

(**D**) Representative examples of injection sites and projection patterns in the dorsal CA1 for CA3- and EC-injected mice. CA3 panels: Coronal section of the hippocampus showing ChR2-eYFP-expressing neurons (green) in CA3 and their axonal projections in dorsal CA1. DAPI staining (white) highlights the hippocampal structure. Axonal projections are localized to the strata oriens and radiatum. EC panels: The left panels show a coronal section of the hippocampus with axonal projections in dorsal CA1 originating from the EC. ChR2-eYFP expression (green) and DAPI staining (white) reveal projections within the strata lacunosum and moleculare. The right panels display a horizontal section of the left EC of the same brain shown on the left, with ChR2-eYFP-expressing neurons (green) and DAPI staining (white). Projections are visible in the strata lacunosum and moleculare of CA1 and the middle molecular layer of the dentate gyrus, consistent with inputs from the medial EC. Scale bars: 200 μm.

Strain	RRID	n	Implant
C57Bl6/J	IMSR_JAX:000664	1	Silicon probe NeuroNexus A1x32-6mm-50-177-H32_21mm
C57Bl6/J	IMSR_JAX:000664	1	Silicon probe NeuroNexus A1x32-5mm-25-177-H32_21mm
C57Bl6/J	IMSR_JAX:000664	1	Silicon probe NeuroNexus A1x64-edge-6mm-20-177-H64LP_30mm
C57Bl6/J	IMSR_JAX:000664	3	Silicon probe Cambridge NeuroTech ASSY-236 H3 Chronic 64-Molex
C57Bl6/J	IMSR_JAX:000664	20	Tetrodes
NDNF-Cre	IMSR_JAX:028536	3	Tetrodes
VIP-Cre	IMSR_JAX:010908	1	Tetrodes
Grik4-Cre	IMSR_JAX:006474	3	Tetrodes
SST-Cre	IMSR_JAX:013044	2	Tetrodes
PV-Cre	IMSR_JAX:008069	6	Tetrodes

Table S1. Details of mouse lines and implants used.

Oscillation	Sub-layer	Mean vector length	Mean Phase (deg)	Nb of cells
**Theta**	**Deep**	0.201 [0.196 – 0.208]	178 [176 – 180]	1634 (1565)
	**Superficial**	0.250 [0.241 – 0.257]	185 [183 – 187]	1333 (1296)
**Slow gamma**	**Deep**	0.184 [0.176 – 0.192]	171 [168 – 175]	385 (381)
	**Superficial**	0.184 [0.168 – 0.202]	178 [173 – 184]	163 (160)
**Mid gamma**	**Deep**	0.102 [0.096 – 0.109]	258 [253 – 262]	320 (226)
	**Superficial**	0.069 [0.064 – 0.075]	263 [257 – 269]	280 (166)
**Rad fast gamma**	**Deep**	0.049 [0.044 – 0.053]	157 [148 – 167]	331 (149)
	**Superficial**	0.041 [0.035 – 0.047]	198 [173 – 229]	87 (31)
**Pyr fast gamma**	**Deep**	0.180 [0.166 – 0.194]	215 [213 – 218]	1041 (724)
	**Superficial**	0.105 [0.088 – 0.124]	226 [218 – 234]	360 (190)
**Ripples**	**Deep**	0.239 [0.226 – 0.252]	201 [199 – 203]	1168 (847)
	**Superficial**	0.129 [0.113 – 0.147]	214 [209 – 220]	460 (240)

Table S2: Oscillatory coupling of CA1 deep and superficial pyramidal sub-layer principal cells. The table shows the mean and 99% bootstrap confidence intervals (in square brackets) for mean vector length and mean phase. Analyses include cells with a minimum of 250 spikes for the given oscillation. In addition, only cells with a mean vector length higher than 0.05 and a p value lower than 0.01 were considered for the mean phase analysis. The 'Nb of cells' column specifies the number of cells included in the mean vector length analysis, with the number for the mean phase analysis indicated in parentheses. Deep cells exhibited significantly stronger coupling to pyramidal fast gamma, mid gamma, radiatum fast gamma, and ripples ($p < 10^{-5}$ for both rate matching and permutation tests in pyramidal fast, mid gamma, and ripples; $p = 0.013$ and $p = 0.0042$ for permutation and rate matching tests in radiatum fast gamma, respectively).
